# First-line Aumolertinib (EGFR tyrosine kinase inhibitor) plus apatinib (VEGFR inhibitor) versus aumolertinib in EGFR-mutant non-small cell lung cancer patients: a randomized, multicenter, phase II trial

**DOI:** 10.1038/s41392-025-02550-y

**Published:** 2026-02-02

**Authors:** Fan Zhang, Zhendong Zheng, Hongmei Zhang, Xiaolong Yan, Zhefeng Liu, Fan Yang, Juyi Wen, Xin Gan, Lin Wu, Shundong Cang, Hongmei Wang, Jun Zhao, Liang Peng, Xiaosong Li, Zaiwen Fan, Ge Shen, Qiong Zhou, Jinjing Zou, Yu Xu, Lei Zhang, Mingfang Zhao, Shangli Cai, Yi Hu

**Affiliations:** 1 Senior Department of Oncology, Chinese PLA General Hospital, Beijing, China; 2 Department of Oncology, General Hospital of the Northern Theater Command of the People’s Liberation Army of China, Liaoning, China; 3 Department of Oncology, The First Affiliated Hospital of Air Force Medical University (Xijing Hospital), Shaanxi, China; 4 Department of Thoracic Surgery, Tangdu Hospital, Air Force Medical University, Shaanxi, China; 5 Department of Medical Oncology, The Third Medical Center, Chinese PLA General Hospital, Beijing, China; 6 Department of Medical Oncology, Sixth Medical Center of PLA General Hospital, Beijing, China; 7 Department of Respiratory and Critical Care Medicine, The First Affiliated Hospital of Nanchang University, Jiangxi, China; 8 Thoracic Medicine Department II, Hunan Cancer Hospital, Hunan, China; 9 Department of Medical Oncology, Henan Provincial People’s Hospital, Henan, China; 10 Department of Respiratory and Critical Care Medicine, The Affiliated Hospital of Qingdao University, Shandong, China; 11 Department of Medical Oncology, Changzhi People’s Hospital, Shanxi, China; 12 Department of Medical Oncology, Fourth Medical Center of PLA General Hospital, Beijing, China; 13 Department of Oncology, Seventh Medical Center of PLA General Hospital, Beijing, China; 14 Department of Oncology, Chinese People’s Liberation Army Air Force Characteristic Medical Center, Beijing, China; 15 Department of Oncology, Beijing Fengtai You’anmen Hospital, Beijing, China; 16 Department of Respiratory and Intensive Care, Union Hospital, Tongji Medical College, Huazhong University of Science and Technology, Hubei, China; 17 Department of Respiratory and Critical Care Medicine, Renmin Hospital of Wuhan University, Hubei, China; 18 Burning Rock Biotech, Guangzhou, China; 19 Department of Medical Oncology, The First Hospital of China Medical University, Liaoning, China

**Keywords:** Drug development, Lung cancer

## Abstract

Inactivating vascular endothelial growth factor receptor (VEGFR) may improve the efficacy of epidermal growth factor receptor (EGFR) tyrosine kinase inhibitors (TKIs) in *EGFR*-mutant non-small cell lung cancer (NSCLC). The ATTENTION study (phase II, open-label, randomized, multicenter trial (Registration number: ChiCTR2100047453), evaluated the efficacy and safety of aumolertinib plus apatinib vs. aumolertinib alone in untreated, *EGFR*-mutant, advanced NSCLC. The primary endpoint was the 18-month PFS rate. Across 18 centers in China, 104 patients were enrolled to receive aumolertinib alone (n = 51) or with apatinib (n = 53). At a median follow-up duration of 19.4 months, aumolertinib plus apatinib outperformed aumolertinib alone in terms of the 18-month progression-free survival (PFS) rate (74% vs. 50%, P = 0.036), median PFS (not reached [NR] vs. 20.1 months, hazard ratio [HR] = 0.41, P = 0.017), and objective response rate (79% vs. 59%, P = 0.024). No grade 4/5 treatment-related adverse effects (TRAEs) were observed, whereas grade 3 TRAEs occurred in 38% vs. 27% of patients, with hypertension (11%) and platelet count decrease (9%) being most common in the combination arm. Exploratory analysis revealed that PFS benefits from aumolertinib plus apatinib predominantly in those with *TP53* mutations. As an infusion-free option, aumolertinib plus apatinib demonstrated PFS benefits with manageable safety in patients with untreated, *EGFR*-mutant, advanced NSCLC.

## Introduction

Lung cancer is the leading cause of cancer-related death worldwide,^1–3^ and nearly 85% of lung cancers are non-small cell lung cancer (NSCLC) subtype.^4^ Epidermal growth factor receptor (EGFR) mutations define a distinct subset of NSCLC, accounting for 47% of the NSCLC cases in the Asia–Pacific region, 15% in Europe, and 22% in North America.^5^ In *EGFR*-mutant advanced NSCLC, tyrosine kinase inhibitors targeting EGFR (EGFR-TKIs) have demonstrated remarkable efficacy, and third-generation EGFR-TKIs have become the preferred first-line option.^6–8^

Despite the ongoing development of novel EGFR-targeting agents, combination strategies based on third-generation EGFR-TKIs as first-line treatments remain an appealing area of research. The addition of chemotherapy, amivantamab, or ramucirumab has been shown to delay recurrence;^9–12^ however, these regimens are often associated with severe adverse effects (AEs) or the requirement for intravenous administration, which can lead to treatment discontinuation and/or reduced adherence and thus limit clinical feasibility.

Multiple randomized controlled trials (RCTs) have reported that adding antiangiogenic agents (bevacizumab, ramucirumab, and apatinib) to first-generation EGFR-TKIs (erlotinib and gefitinib) can confer significant PFS benefits,^13–21^, indicating the feasibility of incorporating antiangiogenic agents in combination therapies.

Aumolertinib, a third-generation EGFR-TKI, has been approved for first-line treatment of advanced *EGFR*-mutated NSCLC patients in China.^22–25^ Here, we conducted this open-label, randomized, multicenter, phase II trial (ATTENTION) to evaluate the efficacy and safety of aumolertinib plus apatinib in untreated patients with *EGFR*-mutant, advanced NSCLC.

## Results

### Patient characteristics

**Fig. 1:**
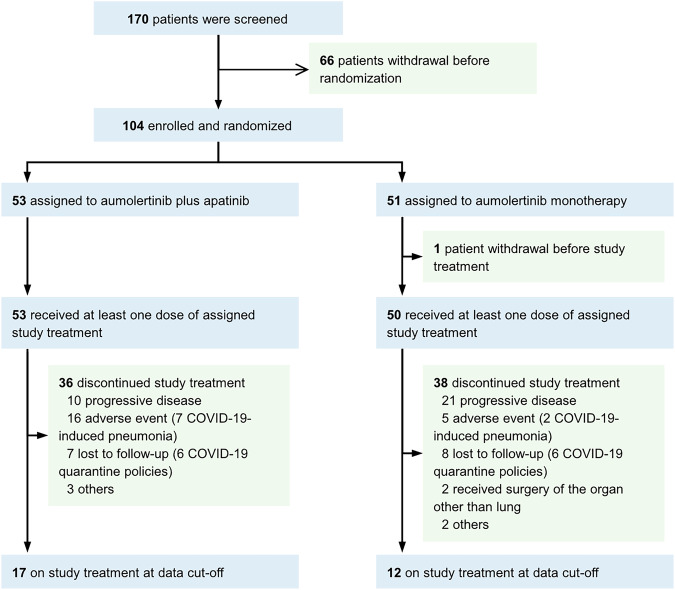
Flow chart of patient selection

The flowchart of the participants is shown in Fig. 1. From June 2021 to November 2022, 104 patients were enrolled and randomized. All patients were included for analysis of treatment efficacy. One patient assigned to the aumolertinib arm withdrew consent before study treatment and was thereby excluded from the safety analysis. Baseline characteristics are shown in Table 1. The median (range) age was 60 (33–75) years. Most of the subjects had adenocarcinoma histology (95%), stage IV disease (95%), and an Eastern Cooperative Oncology Group (ECOG) performance status of 1 (87%). Notably, 26 (25%) patients had central nervous system (CNS) metastasis. With respect to *EGFR* mutations, 50 (48%) and 50 (48%) presented exon 19 deletions and L858R mutations, respectively, and 4 (4%) harbored G719C, L861Q, or G719A mutations, which have been shown to be sensitive to third-generation EGFR-TKIs.^26–28^ The baseline characteristics were generally balanced between the two arms (P > 0.20, Table. 1). Notably, compared to the monotherapy arm, a mildly higher proportion of female was observed in the combination arm (60% vs. 49%, P = 0.24; Table. 1), which requires attention in the subgroup analyses.

**Table 1:** Baseline characteristics

Characteristic	Aumolertinib + Apatinib (N = 53)	Aumolertinib (N = 51)	*P*
Age			0.59
Median (range), y	60 (33–75)	61 (37–75)	
Sex, No. (%)			0.24
Male	21 (40)	26 (51)	
Female	32 (60)	25 (49)	
Smoking history, No. (%)			0.91
Yes	12 (23)	12 (24)	
No	41 (77)	39 (76)	
Histology, No. (%)			0.62
Adenocarcinoma	51 (96)	48 (94)	
Others	2 (4)	3 (6)	
Stage, No. (%)			0.34
IIIB ~ IIIC	1 (2)	4 (8)	
IV	52 (98)	47 (92)	
*EGFR* mutation, No. (%)			0.53
Exon 19 deletion	25 (47)	25 (49)	
L858R	27 (51)	23 (45)	
Others^a^	1 (2)	3 (6)	
ECOG			0.94
0	7 (13)	7 (14)	
1	46 (87)	44 (86)	
Number of metastasis organs, No. (%)			0.91
≥2	14 (26)	14 (27)	
<2	39 (74)	37 (73)	
CNS metastases, No. (%)			0.57
Yes	12 (23)	14 (27)	
No	41 (77)	37 (73)	

*ECOG* Eastern Cooperative Oncology Group

^a^Other EGFR mutations: G719C, G719A and/or L861Q

At the cutoff date (June 20, 2024), the median follow-up was 19.4 months, and 17 of 53 (32%) patients in the aumolertinib‒apatinib arm and 12 of 51 (24%) patients in the aumolertinib arm were still receiving treatment. Notably, 12 patients (12%) discontinued treatment due to the strict quarantine policies of COVID-19 in China, and the discontinuation in 9 cases (9%) was owing to COVID-19-induced pneumonia, leading to a slightly higher rate of non-progression-induced discontinuation than that reported in similar clinical studies.^15,18,29^ Excluding those discontinued treatments, the patients on treatment at the data cutoff were followed up for more than 18 months to ensure the assessment of the 18-month PFS rate (primary endpoint).

### Efficacy

**Fig. 2:**
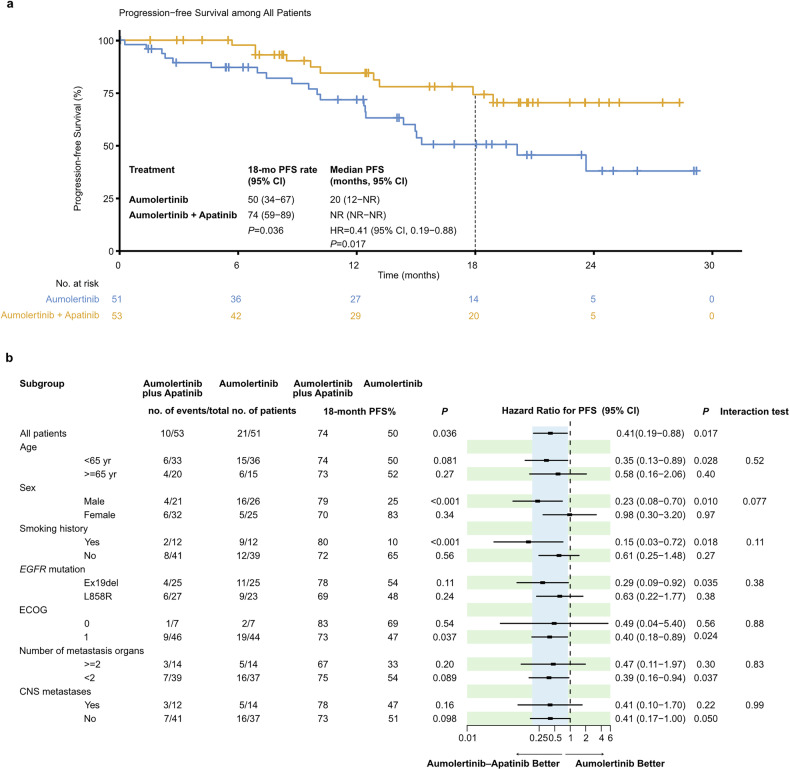
Progression-free survival. **a** Kaplan‒Meier curves of progression-free survival. **b** Subgroup analysis of progression-free survival according to baseline characteristics. CI confidence interval, ECOG Eastern Cooperative Oncology Group, HR hazard ratio, NR not reached

At the data cutoff, progression or death was observed in 10 subjects in the aumolertinib‒apatinib arm (19%) and 21 in the aumolertinib arm (41%). The maturity of overall survival (OS) was 15%, which was insufficient for statistical testing in this interim analysis. The 18-month PFS rate was 74% in patients receiving aumolertinib plus apatinib and greater than 50% in patients treated with aumolertinib monotherapy (P = 0.036; Fig. 2a), meeting the primary endpoint. The median PFS was not reached (NR) in the aumolertinib‒apatinib arm versus 20.1 months in the aumolertinib arm (HR = 0.41, 95% CI: 0.19–0.88, P = 0.017; Fig. 2a). Subgroup analysis for the 18-month PFS rate and PFS revealed comparable benefits in most subgroups, including those with ≥2 metastatic organs, CNS metastasis, and different mutations in *EGFR* (Fig. 2b). Specifically, the addition of apatinib increased the 18-month PFS rate in males (79% vs. 25%) but not in females (70% vs. 83%); a similar trend was observed in the PFS analysis (Fig. 2b). In the multivariable analysis of PFS, the benefit from additional apatinib remained significant after adjusting for sex and other key variables, including age, smoking history, CNS metastasis status, number of metastasis sites, *EGFR* mutation status, and ECOG (HR = 0.38, 95% CI 0.18–0.84, P = 0.016; supplementary Fig. 1).

Since COVID-19 caused the discontinuation of COVID-19 in 21 patients in our study, a sensitivity analysis excluding these patients was performed. The benefits of the 18-month PFS rate and median PFS rate remained significant (18-month PFS rate: 72% vs. 46%, P = 0.025; median PFS: NR vs. 15.0 months, HR = 0.41, 95% CI 0.19–0.86, P = 0.015; Supplementary Fig. 2), indicating that the discontinuation caused by COVID-19 may not significantly affect the study’s conclusions.

**Fig. 3:**
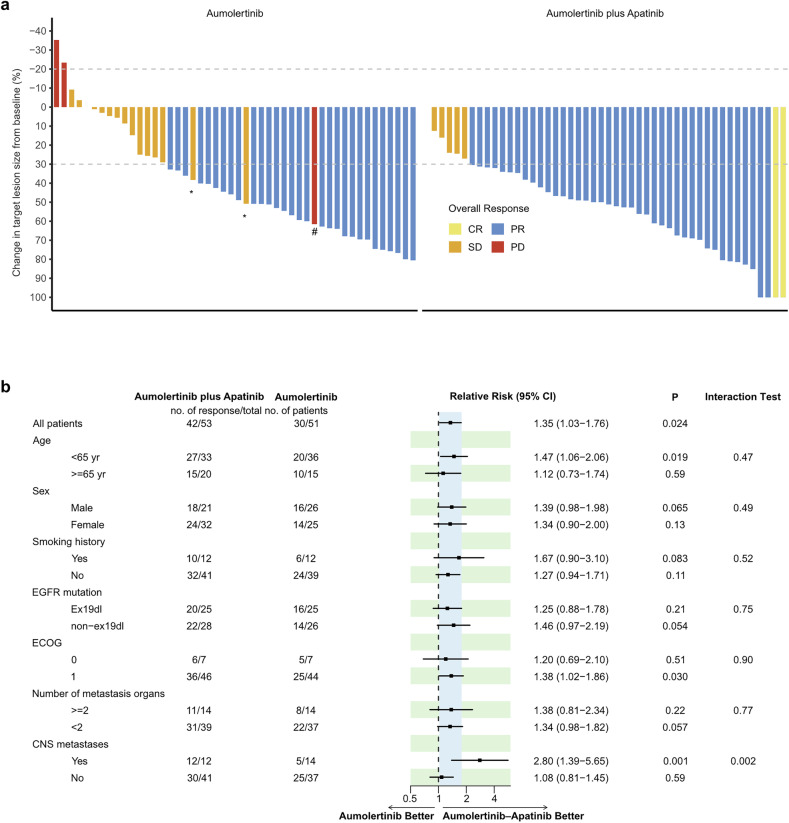
Treatment response assessment. **a** Best percentage change from baseline in target lesion size. # indicates progression of the nontarget lesion. * indicates an unconfirmed PR. **b** Subgroup analysis of the objective response rate according to baseline characteristics. CR complete response, PR partial response, RR risk ratio, SD stable disease, PD progressive disease

The confirmed objective response rate (ORR) was 79% in the aumolertinib‒apatinib arm, which was higher than the 59% in the aumolertinib arm (RR = 1.35, 95% CI 1.03‒1.76, P = 0.024; Table. 2). The detailed depth of the response is shown in Fig. 3a. Time-to-response was similar in the two arms (HR = 1.31, 95% CI 0.80–2.15, P = 0.28; supplementary Fig. 3a), whereas the duration of response (DoR) appeared to be longer in the combination arm (HR = 0.41, 95% CI 0.17–0.99, P = 0.040; supplementary Fig. 3b). The ORR benefit was comparable in most subgroups, while a significant interaction between treatment and CNS metastasis was observed (P = 0.002; Fig. 3b). A remarkable ORR benefit was revealed in patients with CNS metastasis (RR = 2.80, 95% CI 1.39–5.65, P = 0.001), and in those without CNS metastasis, the ORR was similar between the two arms (RR = 1.08, 95% CI 0.81–1.45; P = 0.59; Fig. 3b). Despite this, the DoR in patients without CNS metastasis was better in those who responded to aumolertinib plus apatinib than in those who responded to aumolertinib alone (P = 0.050; supplementary Fig. 3c), indicating the benefit of adding apatinib to these patients. Moreover, the relationship between additional apatinib and a high ORR remained significant in the multivariable logistic regression model (P = 0.015; Supplementary Fig. 4).

**Table 2:** Overall responses

	Aumolertinib + Apatinib (N = 53)	Aumolertinib (N = 51)
Confirmed objective response		
No. of patients with confirmed objective response	42	30
% of patients (95% CI)	79 (66–88)	59 (45–72)
Best overall response–no. (%)		
Complete response	2 (4)	0 (0)
Partial response	40 (76)	30 (59)
Stable disease	6 (11)	15 (29)
Progression disease	0 (0)	3 (6)
Not evaluated	5 (9)	3 (6)
CNS objective response		
No. of patients with CNS metastasis	12	14
No. of patients with CNS objective response	10	6
% of patients (95% CI)	83 (62–100)	43 (17–69)
Time to response–months		
Median (95% CI)	1.6 (1.5–2.9)	1.6 (1.5–2.9)
Duration of response–months		
Median (95% CI)	NR (NR–NR)	22.3 (11.9–NR)

*CI* confidence interval, *NR* not reached

### Safety

The median duration of exposure to apatinib was 8.1 months (interquartile range [IQR]: 3.4–18.9; Supplementary Table 1) in the aumolertinib plus apatinib arm. The median duration of exposure to aumolertinib was 12.8 months (IQR: 6.9–20.9) in the aumolertinib‒apatinib arm and 11.7 months (IQR: 5.7–17.4; Supplementary Table 1) in the aumolertinib arm. Discontinuation with apatinib due to adverse effects was observed in 10 patients (19%) in the aumolertinib-apatinib arm, including pneumonia (n = 3, 6%), pleural effusion (n = 2, 4%), hypertension (n = 1, 2%), rash (n = 1, 2%), proteinuria (n = 1, 2%), hematuresis (n = 1, 2%), and pressure ulcers (n = 1, 2%).

The percentages of patients who experienced any-grade treatment-emergent adverse events (TEAEs) in the aumolertinib-apatinib arm and the aumolertinib arm were 92% and 80%, respectively, and any-grade TRAEs were reported in 91% and 76% of patients (Table 3). Most TRAEs were mild (no grade 4–5 TRAEs), and grade 3 TRAEs were observed in 38% and 27% of patients in the aumolertinib-apatinib arm and the aumolertinib arm, respectively (Table 3). Common grade 3 or worse TRAEs in the aumolertinib‒apatinib arm included hypertension (11%) and decreased platelet count (9%; Table 3).

**Table 3:** Adverse effects

Adverse Events	Aumolertinib + Apatinib (N = 53)			Aumolertinib (N = 51)		
	All Grade	Grade 1-2	Grade 3	All Grade	Grade 1-2	Grade 3
Patients with any TEAE	**49 (92)**		**27 (51)**	**41 (80)**		**19 (37)**
Patients with any TRAE	**48 (91)**		**20 (38)**	**39 (76)**		**14 (27)**
Platelet count decrease	20 (38)	15 (28)	5 (9)	2 (4)	2 (4)	0 (0)
Rash	15 (28)	14 (26)	1 (2)	4 (8)	4 (8)	0 (0)
Diarrhea	14 (26)	14 (26)	0 (0)	4 (8)	2 (4)	2 (4)
Hypertension	14 (26)	8 (15)	6 (11)	1 (2)	0 (0)	1 (2)
CK increased	12 (23)	10 (19)	2 (4)	8 (16)	5 (10)	3 (6)
WBC count decrease	10 (19)	9 (17)	1 (2)	4 (8)	2 (4)	2 (4)
Hypokalaemia	8 (15)	8 (15)	0 (0)	2 (4)	1 (2)	1 (2)
ALT increased	7 (13)	6 (11)	1 (2)	3 (6)	3 (6)	0 (0)
Neutrophil count decrease	7 (13)	6 (11)	1(2)	3 (6)	0 (0)	3 (6)
Anemia	6 (11)	5 (9)	1(2)	1 (2)	1 (2)	0 (0)
Nausea	6 (11)	6 (11)	0 (0)	1 (2)	1 (2)	0 (0)
AST increased	5 (9)	5 (9)	0 (0)	1 (2)	1 (2)	0 (0)
Decreased appetite	5 (9)	4 (7)	1 (2)	1 (2)	1 (2)	0 (0)
Blood bilirubin increased	5 (9)	5 (9)	0 (0)	1 (2)	1 (2)	0 (0)
Insomnia	4 (7)	4 (7)	0 (0)	1 (2)	1 (2)	0 (0)
Weight decreased	4 (7)	4 (7)	0 (0)	1 (2)	0 (0)	1 (2)
Upper respiratory tract infection	4 (7)	4 (7)	0 (0)	2 (4)	2 (4)	0 (0)
GGT increased	3 (6)	2 (4)	1 (2)	1 (2)	0 (0)	1 (2)
Hand-foot syndrome	3 (6)	3 (6)	0 (0)	0 (0)	0 (0)	0 (0)
Headache	3 (6)	2 (4)	1 (2)	0 (0)	0 (0)	0 (0)
Vomiting	2 (4)	2 (4)	0 (0)	2 (4)	2 (4)	0 (0)
Constipation	2 (4)	2 (4)	0 (0)	2 (4)	2 (4)	0 (0)
ECG QT prolonged	2 (4)	1 (2)	1 (2)	1 (2)	0 (0)	1 (2)
Occult blood positive	1 (2)	1 (2)	0 (0)	1 (2)	1 (2)	0 (0)
Abdominal pain	1 (2)	1 (2)	0 (0)	2 (4)	2 (4)	0 (0)

Data in n (%)

*ALT* alanine aminotransferase, *CK* creatine kinase, *ECG* electrocardiograph, *GGT* gamma-glutamyl transpeptidase, *TEAE* treatment emergent adverse effect, *TRAE* treatment-related adverse effect, *WBC* white blood cell

### *TP53* mutations and treatment effects

**Fig. 4:**
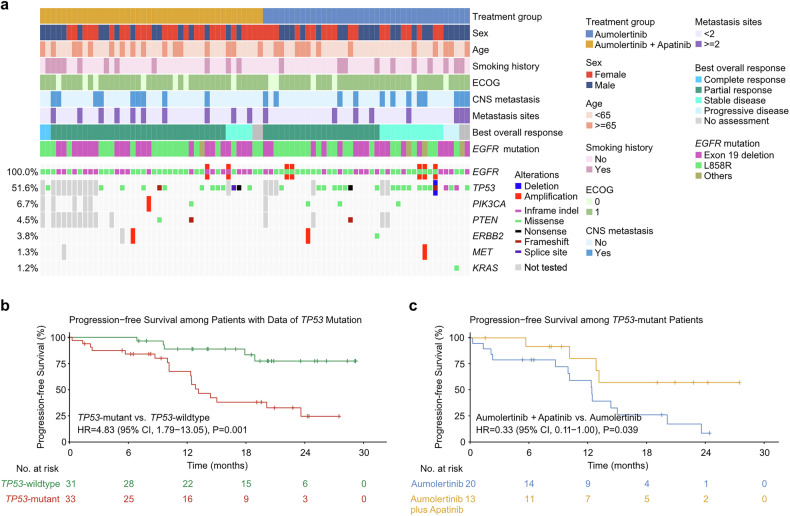
Mutation frequencies and concurrent mutations. **a** Heatmap illustrating the clinical features, treatment response, and mutation landscape of the samples subjected to multigene NGS testing. Kaplan‒Meier curves of progression-free survival between patients with different *TP53* mutation statuses **b** and patients with *TP53* mutation in the two treatment groups **c**. CI confidence interval, HR hazard ratio, NR not reached

Multiple gene polymerase chain reaction (PCR) or next-generation sequencing (NGS) was performed on 42 patients in the aumolertinib‒apatinib arm and 39 patients in the aumolertinib arm. Compared with the overall population, these patients had similar baseline characteristics (Supplementary Table 2). *TP53* was the most mutated tumor-suppressing gene (52%, Fig. 4a**)**, and its mutation was associated with worse PFS (HR = 4.83, 95% CI 1.79–13.05; P = 0.001; Fig. 4b). In the *TP53*-mutant patients with a poorer prognosis, the median PFS was NR in the combination arm and 12.4 months in the monotherapy arm (HR = 0.33, 95% CI 0.11–1.00; P = 0.039; Fig. 4c). In those with wild-type *TP53*, PFS was similar between the two arms (P = 0.30, Supplementary Fig. 5).

## Discussion

ATTENTION demonstrated the superiority of aumolertinib plus apatinib over aumolertinib monotherapy for improving the 18-month PFS rate (74% vs. 50%) and median PFS (NR vs. 20.1 months, HR = 0.41) in untreated patients with *EGFR*-mutant, advanced NSCLC. No grade 4–5 TRAEs were observed, and the incidence rate of grade 3 TRAEs was 38% in the combination arm and 27% in the monotherapy arm. Given this, aumolertinib plus apatinib may serve as a chemotherapy-free and infusion-free option for first-line treatment. Post hoc biomarker analysis revealed the linkage between *TP53* mutation and remarkable benefits from additional apatinib.

Aumolertinib, a third-generation EGFR-TKI, has antitumor efficacy similar to that of osimertinib in both untreated patients and those with a T790M mutation in *EGFR*.^22–25^ The median PFS of patients receiving aumolertinib monotherapy was 20.1 months in our study, closely approximating the 19.3 and 18.9 months reported for patients receiving aumolertinib (AENEAS) and osimertinib monotherapies (FLAURA), respectively.^23,30^ Therefore, the addition of apatinib to aumolertinib further reduced the risk of progression by 59%, potentially making it the optimal choice for first-line therapy. The confirmed ORR in our study of the aumolertinib group was 59%, which was lower than that of the aumolertinib group in the AENEAS study, and the difference might be related to the lower proportion of patients with an *EGFR* exon 19 deletion (proportion of the third-generation EGFR-TKI group in ATTENTION: 49%; AENEAS: 65.4%; FLAURA: 63%).

Subgroup analysis demonstrated that adding apatinib to aumolertinib could confer benefits in most subpopulations. Notably, males and smokers tended to acquire better PFS benefits compared to females and non-smokers, respectively. These results were consistent with a meta-analysis focusing on the impact of adding bevacizumab to osimertinib.^31^

In patients treated with aumolertinib plus apatinib, there were marked increases in the incidence of some adverse effects, e.g., decreased platelet count, rash, diarrhea, and hypertension, which is consistent with previous reports.^18^ Despite these findings, no grade 4–5 TRAEs were reported, and most TRAEs were manageable. The incidence rate of grade 3 or worse TRAEs was 38% in the aumolertinib‒apatinib arm, which was comparable to those in the aumolertinib monotherapy arm (27%). Notably, apatinib-related TRAEs occurred at a lower frequency in the aumolertinib‒apatinib arm than in the CTONG1706 study (gefitinib plus apatinib), which might be due to the relatively lower dosage of apatinib used in our study.^18^ Moreover, the frequency of grade 3 or higher AEs in the aumolertinib‒apatinib arm was markedly lower than that in the osimertinib plus platinum‒pemetrexed chemotherapy arm (64%).^9^ The PFS benefit from the addition of apatinib (HR = 0.41) may be equivalent to that of chemotherapy (HR = 0.62), while chemotherapy can cause severe adverse effects (AEs) and requires intravenous infusion,^9^ which may somewhat affect compliance and clinical applicability. A recent MARIPOSA study demonstrated that the median progression-free survival was significantly longer in the amivantamab–lazertinib group than in the osimertinib group (23.7 vs. 16.6 months), whereas combination therapy failed to increase the objective response rate compared with osimertinib monotherapy, and the rate of grade 3 or higher AEs was 75%.^12^

Loss-of-function mutations of *TP53* reduce its function and thereby relieve its degradation of hypoxia-inducible factor 1 alpha, leading to angiogenesis in tumor xenografts.^32^ In NSCLC tissue samples, *TP53* mutations are independently correlated with high expression of VEGF-A,^33^ suggesting a potential benefit from antiangiogenic agents. Biomarker analyses of RELAY and CTONG1706 also revealed greater PFS benefits from, respectively, adding ramucirumab to erlotinib and adding apatinib to gefitinib in the *TP53*-mutant patients compared to those without.^18,34^ The above findings identified *TP53* mutations as predictive biomarkers of benefits from adding antiangiogenic agents to EGFR-TKIs.

Intriguingly, a phase II RCT in Japan (WJOG9717L) failed, aiming to assess the PFS benefit of adding bevacizumab to osimertinib in the first-line setting (HR = 0.86, one-sided P = 0.21).^29^ This outcome may be attributed to the high objective response rate (ORR) in the control arm (86%), the high discontinuation rate of bevacizumab (64%), and the higher age of enrolled subjects (age ≥ 75 years: 23%).^29^ Other studies evaluating the efficacy of bevacizumab plus osimertinib (NCT04181060, NCT03909334, and TORG1833) are currently ongoing. In contrast to bevacizumab binding exclusively to VEGF, the multitarget apatinib may demonstrate enhanced antitumor efficacy *via* the inhibition of multiple pathways. Furthermore, apatinib can evoke cytotoxic effects, boost autophagy and apoptosis-induced cell death, and optimize the immune microenvironment in vivo;^35,36^ apatinib can also modestly inactivate other tyrosine kinases with the IC_50_ values of 0.013 μM, 0.429 μM, and 0.53 μM for Ret, c-kit, and c-src, respectively.^37^ Whether these functions account for the synergy between apatinib and aumolertinib requires further study.

Our study has several limitations that should be acknowledged. First, ATTENTION is a phase II trial design. Although a significant prolongation of median PFS induced by the addition of apatinib to aumolertinib was revealed, the power of this result is limited by the sample size. The trial was performed in China, with no other ethnicities being treated. Second, COVID-19 and strict quarantine policies in China increased the discontinuation rate in our study, but this effect was comparable between the two arms. Excluding patients who ceased treatment due to COVID-19, those remaining on treatment at the data cutoff were tracked for more than 18 months to ensure a thorough assessment of the 18-month PFS rate (primary endpoint). In addition, the exclusion of these patients did not significantly affect the 18-month PFS rate or median PFS. Third, in our study, some secondary endpoints, including PFS in the treatment group and OS, were immature at the data cutoff for interim analysis, which needs further follow-up.

Aumolertinib plus apatinib may be a compelling candidate therapeutic option for first-line treatment in patients with untreated, *EGFR*-mutant, advanced NSCLC owing to improvements in the ORR and PFS, efficacious disease control in patients with CNS metastasis, a lack of increase in severe TRAEs, and chemo-free and infusion-free characteristics. A phase III trial is warranted to further investigate the efficacy and safety of adding apatinib to aumolertinib.

## Materials and methods

### Study design

ATTENTION is an open-label, randomized, multicenter, phase II trial involving 18 medical centers in China (registration number: ChiCTR2100047453; medical centers: see Supplemental Information, Text S1). The study protocol and amendments were approved by the participating centers’ Ethics Committees (Ethics Number: S2021-233-02). The trial was conducted in compliance with the Declaration of Helsinki. All patients provided informed consent before participation. The complete study protocol can be found in the Supplemental Information (Text S2).

### Participants

The main eligibility criteria were as follows: (1) aged over 18 years; (2) ECOG score of 0 or 1; (3) histologically confirmed treatment-naïve or recurrent stage IIIB–IV NSCLC; (4) harboring *EGFR* exon 19 deletion, L858R mutation, or other *EGFR*-sensitive mutations (e.g., L861Q); and (5) measurable disease according to the Response Evaluation Criteria in Solid Tumors version 1.1 (RECIST v1.1). The key exclusion criteria included previous medical treatment for advanced NSCLC (previous adjuvant or neoadjuvant chemotherapy was allowed if it ended more than 6 months before randomization), symptomatic brain metastases (patients with stable brain metastases are eligible), and known *ALK*/*ROS1* fusions. The detailed inclusion and exclusion criteria are described in the supplemental information (Text S1).

### Randomization and masking

Randomization was performed by stratified blocked randomization (block size: 4) via Clinflash IRT (Clinflash Healthcare Technology (Jiaxing) Co. Ltd., Zhejiang, People’s Republic of China) and stratified by *EGFR* mutation types and CNS metastases. All patients and investigators were unmasked to treatment allocation.

### Procedures

*EGFR* mutations were screened by PCR or NGS using either baseline tumor tissue or plasma samples from hospital laboratories or third-party testing organizations accredited by the Clinical Laboratory Improvement Amendments (CLIA) and the College of American Pathologists (CAP).

The participants were randomly assigned (1:1) to receive either oral aumolertinib (110 mg/day) plus apatinib (250 mg/day) or oral aumolertinib alone (110 mg/day). Therapy was continued until disease progression, unacceptable toxicity, withdrawal of consent, COVID-19-induced pneumonia, or investigator decision. After disease progression, study treatment only continued at the discretion of the investigators. The dose reduction for adverse events of apatinib was permitted to be 250 mg every other day. Patients who discontinued study treatment were followed up for survival until study completion. The criteria for the discontinuation of patients from study participation included the investigator’s decision, becoming pregnant during the study, the patient’s decision to withdraw, or whether the study was stopped for medical, safety, regulatory, or other similar reasons.

Tumor assessments were performed at screening and after 6 weeks (within a window of ±1 week), 12 weeks (window, ±1 week), and then every 6 weeks (window, ±1 week) from randomization until the occurrence of disease progression, as assessed radiologically. Brain scans were performed at screening and at the time of progression in all patients. Patients with brain metastases at screening underwent brain scans at each tumor assessment. The response was reported according to the Response Evaluation Criteria in Solid Tumors, version 1.1 (RECIST 1.1). Treatment-emergent adverse events (TEAEs) were evaluated from the first dose throughout the treatment period and 30 days after the end of treatment according to the Common Terminology Criteria for Adverse Events version 4.0.

NGS or PCR reports illustrating the mutational status of key genes other than *EGFR* in baseline cancer tissue (n = 73) or plasma samples (n = 8) were retrieved to explore biomarkers. The panels used are shown in the Supplemental Information (Text S1).

### Outcomes

The primary endpoint was the 18-month PFS rate, defined as the proportion of patients at 18 months after randomization who were alive and had not experienced disease progression (assessed by RECIST v1.1). Patients with treatment discontinuation for any reason were censored at the date of the last progression assessment with no documented progression. Those on treatment at the data cutoff should have been tracked for more than 18 months.

The secondary endpoints consisted of PFS (time from randomization to the first time of disease progression or death), confirmed ORR (the proportion of patients with confirmed complete response or partial response), DoR (time from the first documented response to the first time of disease progression or death), safety, and OS (time from randomization to death). Exploratory endpoints included PFS benefits in patients with CNS metastasis, *EGFR* exon 19 deletion, *EGFR* L858R mutation, and other mutational events.

### Sample size calculation

This trial aimed to observe the superiority of aumolertinib plus apatinib over aumolertinib alone. Given the assumptions of an 18-month PFS rate of 75% for aumolertinib plus apatinib and 50% for aumolertinib alone^23^ and a 10% drop-out rate, to achieve 80% power at the 5% level of significance with equal allocation, a total of 98 subjects were needed (49 per arm).^38^

### Statistical analysis

To assess the between-group differences, we used (i) the chi-square test for categorical variables, (ii) the Mann‒Whitney test for continuous variables, (iii) the Kaplan‒Meier method and the log-rank test for time‒event variables, and (iv) the “naïve” test, which is based on the comparison of Kaplan‒Meier estimators for the 18-month PFS rate (“Fixpoint.test” function of the R package: ComparisonSurv).^39^ Hazard ratios (HRs) and corresponding 95% confidence intervals (CIs) were estimated via the Cox proportional hazards model. The 95% CIs for the ORR were generated through the Clopper‒Pearson method. Univariable and multivariable analyses of PFS and ORR were performed with the Cox proportional hazards model and the logistic regression model, respectively. The interaction effect between treatment and each key variable was calculated via a Cox proportional hazards model (for PFS) or a logistic regression model (for ORR) that included the treatment group, key variable, and treatment-by-variable interaction term.^40^ All the statistical analyses mentioned above were performed via IBM SPSS Statistics 22 or R 4.1.3. The complete statistical analysis plan can be found in the Supplemental Information (Text S2).

## Supplementary information

Supplementary Materials

study protocol

## Data Availability

The study protocol, statistical analysis plan, and related materials are publicly available. The datasets generated and analyzed are not publicly available owing to ethical and regulatory constraints, but can be obtained from the corresponding authors upon reasonable request. Deidentified participant data may be shared, subject to the approval of a proposal, which must detail the study objectives and statistical analysis plan, by all corresponding authors.

## References

[1] Siegel, R. L., Miller, K. D., Wagle, N. S. & Jemal, A. Cancer statistics, 2023. *CA Cancer J. Clin.* **73**, 17–48 (2023). 36633525 10.3322/caac.21763

[2] He, S. et al. Cancer profiles in China and comparisons with the USA: a comprehensive analysis in the incidence, mortality, survival, staging, and attribution to risk factors. *Sci. China Life Sci.* **67**, 122–131 (2024). 37755589 10.1007/s11427-023-2423-1

[3] Gatta, G. et al. Burden and centralized treatment in Europe of rare tumors: results of RARECAREnet—a population-based study. *Lancet Oncol.* **18**, 1022–1039 (2017). 28687376 10.1016/S1470-2045(17)30445-X

[4] Gridelli, C. et al. Non-small cell lung cancer. *Nat. Rev. Dis. Prim.* **1**, 15009 (2015). 27188576 10.1038/nrdp.2015.9

[5] Midha, A., Dearden, S. & McCormack, R. EGFR mutation incidence in non-small cell lung cancer of adenocarcinoma histology: a systematic review and global map by ethnicity (mutMapII). *Am. J. Cancer Res.* **5**, 2892–2911 (2015). 26609494 PMC4633915

[6] Hendriks, L. E. et al. Oncogene-addicted metastatic non-small cell lung cancer: ESMO Clinical Practice Guideline for diagnosis, treatment and follow-up. *Ann. Oncol.* **34**, 339–357 (2023). 36872130 10.1016/j.annonc.2022.12.009

[7] Riely, G. J. et al. Non-small cell lung cancer, version 4.2024, NCCN clinical practice guidelines in oncology. *J. Natl. Compr. Cancer Netw.* **22**, 249–274 (2024). 10.6004/jnccn.2204.0023 38754467

[8] Medical Oncology Branch of China International, E. Promotive Association for, M., Health, C. & Chinese Association for Clinical, O. [China clinical practice guideline for epidermal growth factor receptor tyrosine kinase inhibitors in stage Ⅳ non-small cell lung cancer (version 2023)]. *Zhonghua. Yi Xue. Za Zhi.* **103**, 3160–3173 (2023). 10.3760/cma.j.cn112137-20230505-00725 37879869

[9] Planchard, D. et al. Osimertinib with or without chemotherapy in EGFR-mutated advanced NSCLC. *N. Engl. J. Med.* **389**, 1935–1948 (2023). 37937763 10.1056/NEJMoa2306434

[10] Felip, E. et al. Amivantamab plus lazertinib versus osimertinib in first-line EGFR-mutant advanced non-small cell lung cancer with biomarkers of high-risk disease: a secondary analysis from MARIPOSA. *Ann. Oncol.* **35**, 805–816 (2024). 38942080 10.1016/j.annonc.2024.05.541

[11] Le, X. et al. A Multicenter open-label randomized phase ii study of osimertinib with and without ramucirumab in tyrosine kinase inhibitor-naïve EGFR-mutant metastatic non-small cell lung cancer (RAMOSE trial). *J. Clin. Oncol*. **43**, 403–411 (2024). 10.1200/JCO.24.00533 PMC11776886 39378386

[12] Cho, B. C. et al. Amivantamab plus lazertinib in previously untreated EGFR-mutated advanced NSCLC. *N. Engl. J. Med.* **391**, 1486–1498 (2024). 10.1056/NEJMoa2403614 38924756

[13] Saito, H. et al. Erlotinib plus bevacizumab versus erlotinib alone in patients with EGFR-positive advanced nonsquamous non-small cell lung cancer (NEJ026): interim analysis of an open-label, randomized, multicenter, phase 3 trial. *Lancet Oncol.* **20**, 625–635 (2019). 30975627 10.1016/S1470-2045(19)30035-X

[14] Zhou, Q. et al. Bevacizumab plus erlotinib in Chinese patients with untreated, EGFR-mutated, advanced NSCLC (ARTEMIS-CTONG1509): a multicenter phase 3 study. *Cancer Cell* **39**, 1279–1291 e1273 (2021). 34388377 10.1016/j.ccell.2021.07.005

[15] Nakagawa, K. et al. Ramucirumab plus erlotinib in patients with untreated, EGFR-mutated, advanced non-small cell lung cancer (RELAY): a randomized, double-blind, placebo-controlled, phase 3 trial. *Lancet Oncol.* **20**, 1655–1669 (2019). 31591063 10.1016/S1470-2045(19)30634-5

[16] Yamamoto, N. et al. Erlotinib plus bevacizumab vs erlotinib monotherapy as first-line treatment for advanced EGFR mutation-positive nonsquamous non-small cell lung cancer: Survival follow-up results of the randomized JO25567 study. *Lung Cancer* **151**, 20–24 (2021). 33279874 10.1016/j.lungcan.2020.11.020

[17] Seto, T. et al. Erlotinib alone or with bevacizumab as first-line therapy in patients with advanced nonsquamous non-small cell lung cancer harboring EGFR mutations (JO25567): an open-label, randomized, multicenter, phase 2 study. *Lancet Oncol.* **15**, 1236–1244 (2014). 25175099 10.1016/S1470-2045(14)70381-X

[18] Zhao, H. et al. Apatinib plus gefitinib as first-line treatment in advanced EGFR-mutant NSCLC: the phase III ACTIVE study (CTONG1706). *J. Thorac. Oncol.* **16**, 1533–1546 (2021). 34033974 10.1016/j.jtho.2021.05.006

[19] Kawashima, Y. et al. Bevacizumab plus erlotinib versus erlotinib alone in Japanese patients with advanced, metastatic, EGFR-mutant non-small cell lung cancer (NEJ026): overall survival analysis of an open-label, randomized, multicenter, phase 3 trial. *Lancet Respir. Med.* **10**, 72–82 (2022). 34454653 10.1016/S2213-2600(21)00166-1

[20] Stinchcombe, T. E. et al. Effect of erlotinib plus bevacizumab vs erlotinib alone on progression-free survival in patients with advanced EGFR-mutant non-small cell lung cancer: a phase 2 randomized clinical trial. *JAMA Oncol.* **5**, 1448–1455 (2019). 31393548 10.1001/jamaoncol.2019.1847 PMC6692685

[21] Piccirillo, M. C. et al. Addition of bevacizumab to erlotinib as first-line treatment of patients with EGFR-mutated advanced nonsquamous NSCLC: the BEVERLY multicenter randomized phase 3 trial. *J. Thorac. Oncol.* **17**, 1086–1097 (2022). 35659580 10.1016/j.jtho.2022.05.008

[22] Lu, S. et al. Central nervous system efficacy of aumolertinib versus gefitinib in patients with untreated, EGFR-mutated, advanced non-small cell lung cancer: data from a randomized phase III trial (AENEAS). *Cancer Commun.* **44**, 1005–1017 (2024). 10.1002/cac2.12594 PMC11492357 39016053

[23] Lu, S. et al. AENEAS: a randomized phase III trial of aumolertinib versus gefitinib as first-line therapy for locally advanced or metastatic non-small cell lung cancer with EGFR Exon 19 deletion or L858R mutations. *J. Clin. Oncol.* **40**, 3162–3171 (2022). 35580297 10.1200/JCO.21.02641 PMC9509093

[24] Lu, S. et al. Efficacy of aumolertinib (HS-10296) in patients with advanced EGFR T790M+ NSCLC: updated post-national medical products administration approval results from the APOLLO registrational trial. *J. Thorac. Oncol.* **17**, 411–422 (2022). 34801749 10.1016/j.jtho.2021.10.024

[25] Nagasaka, M. et al. Beyond osimertinib: the development of third-generation EGFR tyrosine kinase inhibitors for advanced EGFR+ NSCLC. *J. Thorac. Oncol.* **16**, 740–763 (2021). 33338652 10.1016/j.jtho.2020.11.028

[26] Shi, C. et al. Antitumor activity of aumolertinib, a third-generation EGFR tyrosine kinase inhibitor, in non-small cell lung cancer harboring uncommon EGFR mutations. *Acta Pharm. Sin. B.* **13**, 2613–2627 (2023). 37425047 10.1016/j.apsb.2023.03.007 PMC10326255

[27] Li, L. Two non-small cell lung cancer (NSCLC) patients with brain metastasis harboring epidermal growth factor receptor (EGFR) G719X and L861Q mutations benefited from aumolertinib: two cases report and review of the literature. *Heliyon* **8**, e10407 (2022). 36119883 10.1016/j.heliyon.2022.e10407 PMC9474834

[28] Cho, J. H. et al. Osimertinib for patients with non-small cell lung cancer harboring uncommon EGFR mutations: a multicenter, open-label, phase II trial (KCSG-LU15-09). *J. Clin. Oncol.* **38**, 488–495 (2020). 31825714 10.1200/JCO.19.00931 PMC7098834

[29] Kenmotsu, H. et al. Randomized phase 2 study of osimertinib plus bevacizumab versus osimertinib for untreated patients with nonsquamous NSCLC harboring EGFR mutations: WJOG9717L study. *J. Thorac. Oncol.* **17**, 1098–1108 (2022). 35636696 10.1016/j.jtho.2022.05.006

[30] Soria, J. C. et al. Osimertinib in untreated EGFR-mutated advanced non-small cell lung cancer. *N. Engl. J. Med.* **378**, 113–125 (2018). 29151359 10.1056/NEJMoa1713137

[31] Zhou, G., Guo, L., Xu, J., Tang, K. & Chen, J. Comparison of osimertinib plus bevacizumab against osimertinib alone in NSCLC harboring EGFR mutations: a systematic review and meta-analysis. *Ther. Adv. Med. Oncol.* **16**, 17588359241227677 (2024). 38304850 10.1177/17588359241227677 PMC10832416

[32] Ravi, R. et al. Regulation of tumor angiogenesis by p53-induced degradation of hypoxia-inducible factor 1alpha. *Genes Dev.* **14**, 34–44 (2000). 10640274 PMC316350

[33] Schwaederle, M. et al. VEGF-a expression correlates with TP53 mutations in non-small cell lung cancer: implications for antiangiogenesis therapy. *Cancer Res.* **75**, 1187–1190 (2015). 25672981 10.1158/0008-5472.CAN-14-2305

[34] Garon, E. B. et al. Ramucirumab plus erlotinib versus placebo plus erlotinib in previously untreated EGFR-mutated metastatic non-small cell lung cancer (RELAY): exploratory analysis of next-generation sequencing results. *ESMO Open* **8**, 101580 (2023). 37390764 10.1016/j.esmoop.2023.101580 PMC10485403

[35] Xie, C. et al. Apatinib triggers autophagic and apoptotic cell death via VEGFR2/STAT3/PD-L1 and ROS/Nrf2/p62 signaling in lung cancer. *J. Exp. Clin. Cancer Res.* **40**, 266 (2021). 34429133 10.1186/s13046-021-02069-4 PMC8385858

[36] Zhao, S. et al. Low-dose apatinib optimizes tumor microenvironment and potentiates antitumor effect of PD-1/PD-L1 blockade in lung cancer. *Cancer Immunol. Res.* **7**, 630–643 (2019). 30755403 10.1158/2326-6066.CIR-17-0640

[37] Tian, S. et al. YN968D1 is a novel and selective inhibitor of vascular endothelial growth factor receptor-2 tyrosine kinase with potent activity in vitro and in vivo. *Cancer Sci.* **102**, 1374–1380 (2011). 21443688 10.1111/j.1349-7006.2011.01939.x PMC11158267

[38] Wang, X. & Ji, X. Sample size estimation in clinical research: from randomized controlled trials to observational studies. *Chest* **158**, S12–S20 (2020). 32658647 10.1016/j.chest.2020.03.010

[39] Klein, J. P., Logan, B., Harhoff, M. & Andersen, P. K. Analyzing survival curves at a fixed point in time. *Stat. Med.* **26**, 4505–4519 (2007). 17348080 10.1002/sim.2864

[40] Ballman, K. V. Biomarker: predictive or prognostic? *J. Clin. Oncol.* **33**, 3968–3971 (2015). 26392104 10.1200/JCO.2015.63.3651

