# Supplementary material for: First-line Aumolertinib (EGFR tyrosine kinase inhibitor) plus apatinib (VEGFR inhibitor) versus aumolertinib in EGFR-mutant non-small cell lung cancer patients: a randomized, multicenter, phase II trial
Source: Signal Transduct Target Ther. 2026 Feb 2;11:40. doi: 10.1038/s41392-025-02550-y (PMC12864929; doi:10.1038/s41392-025-02550-y)
Supplement: Supplementary file 1 — Supplementary Materials [file 41392_2025_2550_MOESM1_ESM.docx]

Supplementary Materials for

Aumolertinib plus apatinib versus aumolertinib in EGFR-mutant NSCLC patients

Fan Zhang, Zhendong Zheng, Hongmei Zhang, Xiaolong Yan, Zhefeng Liu, Fan Yang, Juyi Wen, Xin Gan, Lin Wu, Shundong Cang, Hongmei Wang, Jun Zhao, Liang Peng, Xiaosong Li, Zaiwen Fan, Ge Shen, Qiong Zhou, Jinjing Zou, Yu Xu, Lei Zhang, Mingfang Zhao, Shangli Cai, Yi Hu

Correspondence to: Yi Hu, [huyi301zlxb@sina.com](mailto:huyi301zlxb@sina.com); Shangli Cai, [shangli.cai@brbiotech.com](mailto:shangli.cai@brbiotech.com); Mingfang Zhao, [zhaomf618@126.com](mailto:zhaomf618@126.com)

**This PDF file includes:**

Materials

Figures. S1 to S6

Tables S1 to S2

Figure. S1. Univariable and multivariable analyses of progression-free survival.


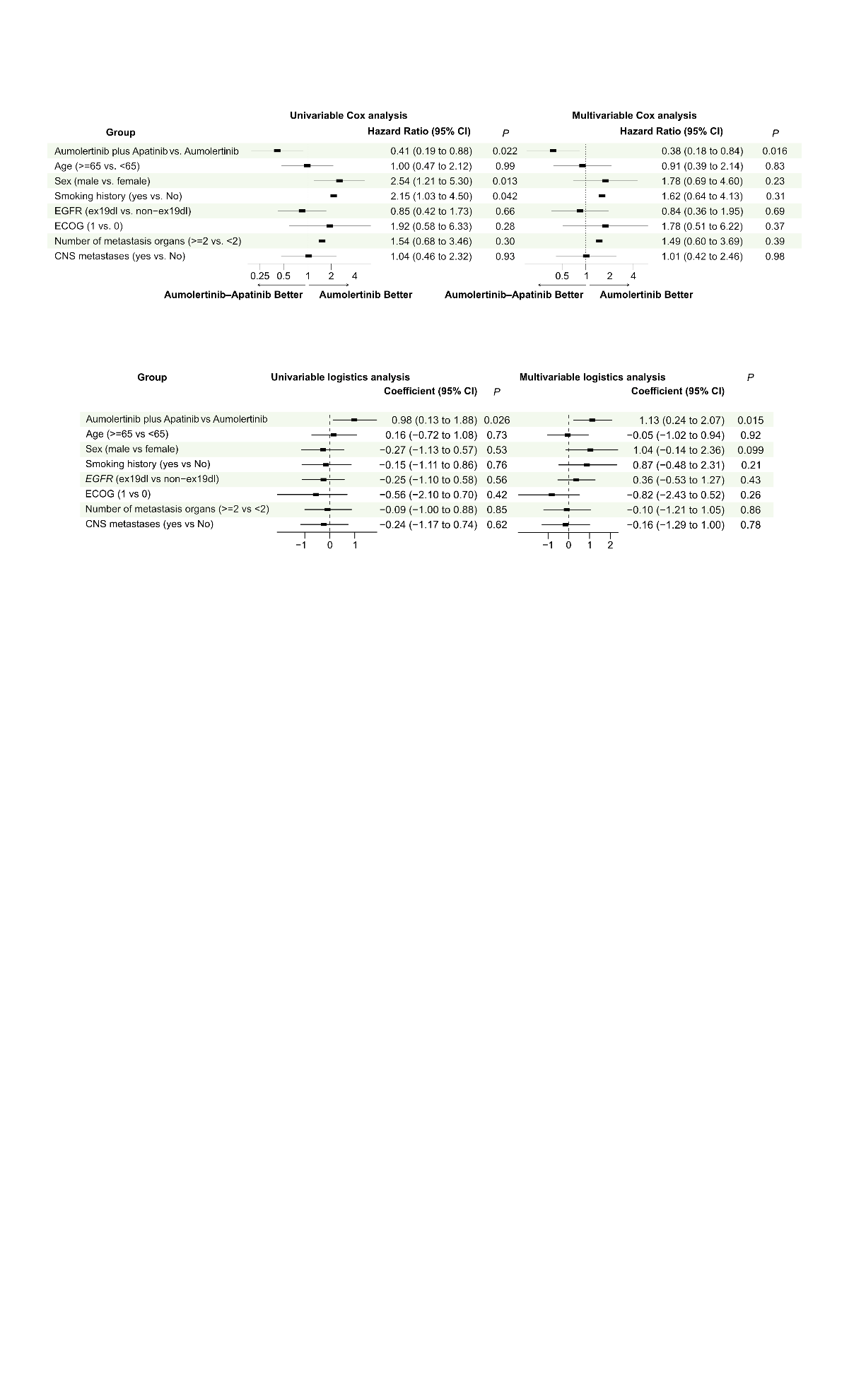


Univariable and multivariable analyses of progression-free survival according to baseline characteristics. CI, confidence interval; CNS, central nervous system; ex19del, exon 19 deletion; ECOG, Eastern Cooperative Oncology Group.


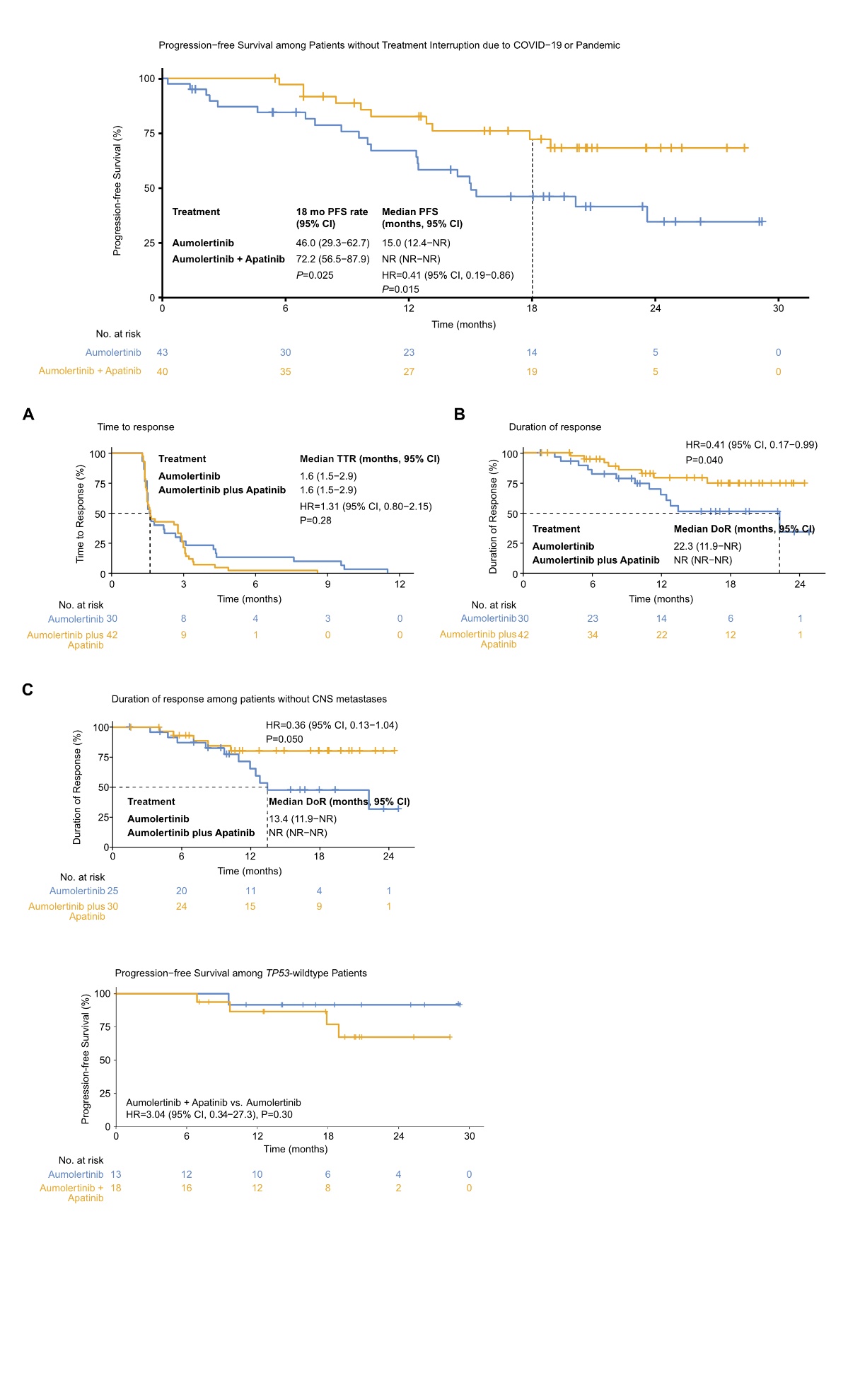
Figure. S2. Progression-free survival in patients who did not discontinue treatment due to COVID-19.

Kaplan‒Meier curves of progression-free survival in patients who did not discontinue treatment due to COVID-19.

Figure. S3. Time to response and duration of response.


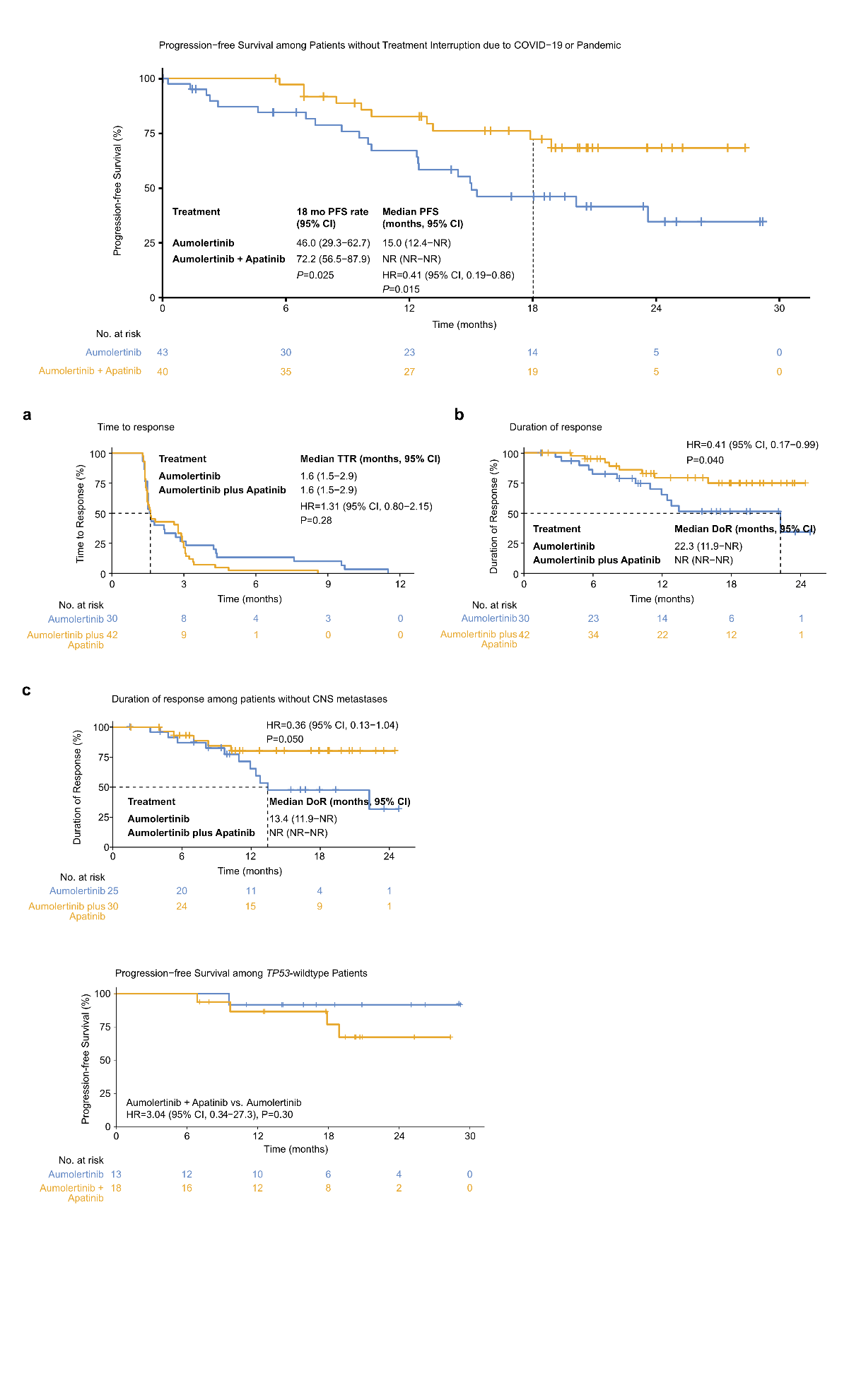


(**a**) Time to response in patients treated with aumolertinib plus apatinib or aumolertinib alone. (**b-c**) Duration of response in the aumolertinib-apatinib arm and the aumolertinib monotherapy arm of all patients (**b**) or patients without CNS metastases (**c**).

Figure. S4. Univariable and multivariable analyses of objective response rate.


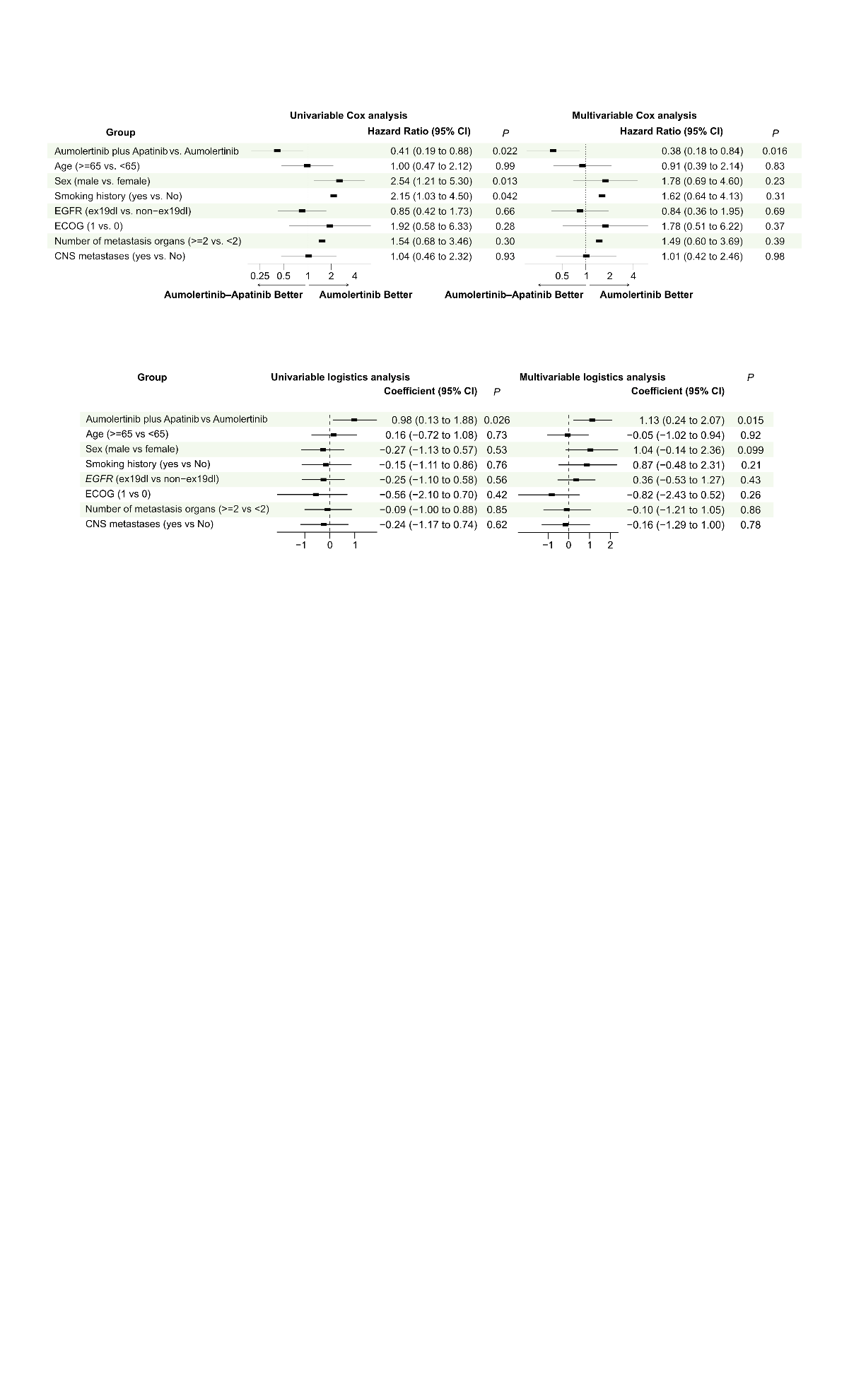


Univariable and multivariable analyses of objective response rate according to baseline characteristics. CI, confidence interval; ex19del, exon 19 deletion; ECOG, Eastern Cooperative Oncology Group.


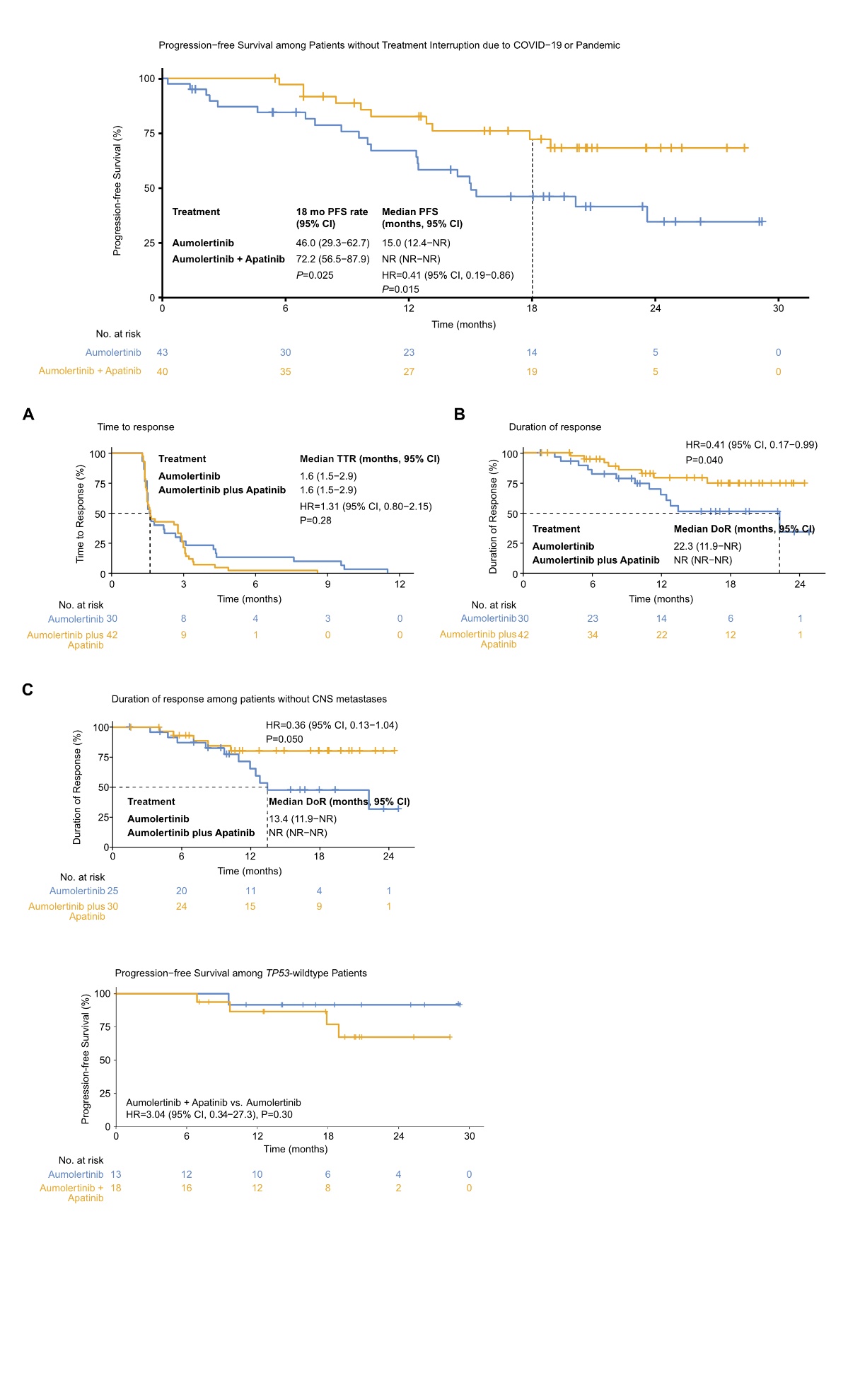
Figure. S5. Progression-free survival on aumolertinib alone or with apatinib among patients without *TP53* mutations.

Kaplan‒Meier curves of progression-free survival in patients without *TP53* mutant patients in two treatment group. CI, confidence interval; HR, hazard ratio; NR, not reached.

Table S1. Treatment exposure

|  | **Aumolertinib plus Apatinib (N=53)** | **Aumolertinib (N=51)** |
| --- | --- | --- |
|  | N=53 | N=51 |
| **Treatment duration of aumolertinib** |  |  |
| Median (range), months | 12.8 (6.9-20.9) | 11.7 (5.7-17.4) |
| Treatment duration of apatinib |  |  |
| Median (range), months | 8.1 (3.4-18.9) | - |
| **Dose interruption with aumolertinib — no.** |  |  |
| Diarrhea | 1 | 0 |
| Interstitial pneumonia | 1 | 0 |
| Hepatic toxicity | 0 | 1 |
| **Dose interruption with apatinib — no.** | **13** |  |
| Hypertension | 4 | - |
| PLT decrease | 3 | - |
| CK increase | 1 | - |
| ECG QT prolonged | 1 | - |
| Diarrhea | 1 | - |
| Headache | 1 | - |
| WBC decrease | 1 | - |
| Reasons other than AEs | 1 | - |
| **Discontinuation with study treatment — no.** | 36 | 38 |
| Disease progression | 10 | 21 |
| AEs | 16 (7 cases were due to COVID-19-induced pneumonia) | 5 (2 cases were due to COVID-19-induced pneumonia) |
| Lost to follow-up | 7 (6 cases were due to COVID-19 quarantine policies) | 8 (6 cases were due to COVID-19 quarantine policies) |
| Receiving operation of other organs | 0 | 2 |
| Others | 3 | 2 |
| **Discontinuation with apatinib — no.** | 10 | - |
| Pneumonia | 3 | - |
| Pleural effusion | 2 | - |
| Hypertension | 1 | - |
| Rash | 1 | - |
| Proteinuria | 1 | - |
| Hematuresis | 1 | - |
| Pressure ulcer | 1 |  |
| **Dose reduction with apatinib — no. (include overlap)** | 18 | - |
| Diarrhea | 3 | - |
| Hypertension | 3 | - |
| PLT decrease | 3 | - |
| Rash | 2 | - |
| Headache | 1 | - |
| Proteinuria | 1 | - |
| Peripheral edema | 1 | - |
| Reasons other than AEs | 8 | - |

Table S2. Baseline characteristics of all patients and those with genomic data.

|  | All patients | Patients with genomic data | P |
| --- | --- | --- | --- |
| n | 104 | 81 |  |
| Treatment (%) |  |  | 0.90 |
| Aumolertinib plus Apatinib | 53 (51) | 42 (52) |  |
| Aumolertinib | 51 (49) | 39 (48) |  |
| Age (%) |  |  | 0.90 |
| >=65 | 35 (34) | 28 (35) |  |
| <65 | 69 (66) | 53 (65) |  |
| Sex (%) |  |  | 0.82 |
| Male | 47 (45) | 38 (47) |  |
| Female | 57 (55) | 43 (53) |  |
| Smoking history (%) |  |  | 0.65 |
| Yes | 24 (23) | 21 (26) |  |
| No | 80 (77) | 60 (74) |  |
| Histology (%) |  |  | 1.00 |
| Lung adenocarcinoma | 99 (95) | 77 (95) |  |
| others | 5 (5) | 4 (5) |  |
| EGFR mutation (%) |  |  | 0.93 |
| Ex19del | 50 (48) | 38 (47) |  |
| L858R | 50 (48) | 39 (48) |  |
| others | 4 (4) | 4 (5) |  |
| ECOG (%) |  |  |  |
| 1 | 90 (87) | 68 (84) | 0.62 |
| 0 | 14 (13) | 13 (16) |  |
| Number of metastasis organs (%) |  |  | 0.54 |
| >=2 | 28 (27) | 18 (22) |  |
| <2 | 76 (73) | 63 (78) |  |
| CNS metastases (%) |  |  | 0.89 |
| Yes | 26 (25) | 21 (26) |  |
| No | 78 (75) | 60 (74) |  |

CNS, central nervous system; Ex19del, exon 19 deletion; ECOG, Eastern Cooperative Oncology Group.
