# Supplementary material for: First-line Aumolertinib (EGFR tyrosine kinase inhibitor) plus apatinib (VEGFR inhibitor) versus aumolertinib in EGFR-mutant non-small cell lung cancer patients: a randomized, multicenter, phase II trial
Source: Signal Transduct Target Ther. 2026 Feb 2;11:40. doi: 10.1038/s41392-025-02550-y (PMC12864929; doi:10.1038/s41392-025-02550-y)
Supplement: Supplementary file 2 — study protocol [file 41392_2025_2550_MOESM2_ESM.pdf]

Chinese PLA General Hospital (301 Military Hospital)

Chinese Clinical Trial Registry Number: ChiCTR2100047453

Aumolertinib plus apatinib versus aumolertinib as first-line treatment in patients with *EGFR* mutation positive locally advanced or metastatic non-small cell lung cancer (NSCLC): a randomized, multicenter study

Indication: Locally advanced or metastatic non-small cell lung cancer

Sponsor: Chinese PLA General Hospital (301 Military Hospital)

Address: No. 28, Fuxing Road, Haidian District, Beijing, China

Version: 3.0

Version Date: 01 June 2021

Confidentiality Statement

The information contained in this protocol is confidential and belongs to Chinese PLA General Hospital (301 Military Hospital). It may not be used or disclosed without authorization

TABLE OF CONENTS

1. Administrative Information 3

1.1 Contact Information 3

1.2 Signature Page 4

2. Synopsis 5

3. Table of Abbreviations 12

4. Research Background and Scientific Basis 15

4.1 Background 15

4.2 Scientific Evidence 17

5. Study Endpoints 20

6. Sample Size 20

7. Study Design and Description 21

7.1 Study Design 21

7.2 Inclusion Criteria 21

7.3 Exclusion Criteria 23

7.4 Randomization 26

8. Study Procedures 27

8.1 Screening Period 27

8.2 Treatment Period 27

8.3 Follow-up Period 28

9. Endpoint Evaluation and Statistical Analysis 28

9.1 Anti-Tumor Activity Evaluation 28

9.1.1 Investigator Assessment as per RECIST 1.1 28

9.1.2 18-month Progression-free Survival (PFS) Rate 29

9.1.3 Overall survival (OS) 29

9.1.4 Confirmed Objective Response Rate (ORR) 29

9.1.5 Duration of Response (DoR) 30

9.2 Safety Evaluation 31

9.3 Recommended Toxicity Management 31
10. Statistical Analytical Methods 31
10.1 Efficacy Analysis 32
10.1.1 Analysis of Primary Efficacy Endpoint (18-month PFS rate) 32
10.1.2 Analysis of Secondary Efficacy Endpoints 32
10.1.3 Analysis of Exploratory Endpoints 32
10.1.4 Time of Interim Analysis 32
10.1.5 Time of OS Analysis 33
10.2 Safety Analysis 33
10.2.1 Adverse Events 33
10.2.2 Independent Data Monitoring Committee (IDMC) 33
References 34
Appendix A Study Plan 36

1. Administrative Information

1.1 Contact Information

Separate contact information will be provided for each study center. An emergency medical contact information card will be provided for each subject.

Clinical trial site details

**Investigator**	**Department**	**Medical Institution**
Yi Hu	Oncology	The First Medical Center, Chinese PLA General Hospital.
Mingfang Zhao	Medical Oncology	The First Hospital of China Medical University
Zhendong Zheng	Oncology	General Hospital of Northern Theater Command.
Hongmei Zhang	Oncology	Xijing Hospital of Air Force Military Medical University
Xiaolong Yan	Thoracic Surgery	Tangdu Hospital, Air Force Medical University
Zhefeng Liu	Medical Oncology	The Third Medical Center, Chinese PLA General Hospital.
Juyi Wen	Medical Oncology	Sixth Medical Center of PLA General Hospital.
Xin Gan	Respiratory and Critical Care Medicine	The First Affiliated Hospital of Nanchang University,
Lin Wu	Thoracic Medicine Department II	Hunan Cancer Hospital .
Shundong Cang	Department of Medical Oncology	Henan Provincial People's Hospital
Hongmei Wang	Respiratory and Critical Care Medicine	The Affiliated Hospital of Qingdao University
Jun Zhao	Department of Medical Oncology	Changzhi People's Hospital.
Liang Peng	Department of Medical Oncology	Fourth Medical Center of PLA General Hospital.
Xiaosong Li	Department of Oncology	Seventh Medical Center of PLA General Hospital.
Zaiwen Fan	Department of Oncology	Chinese People's Liberation Army Air Force Characteristic Medical Center
Ge Shen	Department of Oncology	Beijing Fengtai You'anmen Hospital
Qiong Zhou	Department of	Union Hospital, Tongji Medical College, Huazhong

	Respiratory and Intensive Care	University of Science and Technology
Jinjing Zou	Respiratory and Critical Care Medicine	Renmin Hospital of Wuhan University

1.2 Signature Page

Chinese PLA General Hospital (301 Military Hospital)

With the utmost respect for the personnel participating in the trial, the trial will be carried out in accordance with the protocol and the following:

- The ethical principles of the Declaration of Helsinki
- ICH E6 GCP- Good Clinical Practice
- All applicable laws and regulations, including but not limited to laws and regulations on data confidentiality and disclosure of clinical trial information

Signatures

____

Leading Investigator's Signature

Date

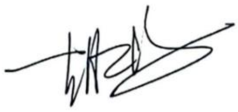

01 June 2021

Director of the Leadership Unit

Chinese PLA General Hospital (301 Military Hospital)

Adress

No. 28, Fuxing Road ,Haidian District, Beijing, China

2. Synopsis

<table>
<tr><td>Sponsor: Chinese PLA General Hospital (301 Military Hospital)</td><td>Study Drug: aumolertinib, apatinib</td></tr>
<tr><td>Title of Protocol: Aumolertinib plus apatinib versus aumolertinib as first-line treatment in patients with EGFR mutation positive locally advanced or metastatic non-small cell lung cancer (NSCLC): a randomized, multicenter study</td><td>EudraCT Number: N/A</td></tr>
<tr><td>Study Number: ChiCTR2100047453</td><td>Study Phase: Phase II</td></tr>
<tr><td colspan="2">Study Design:
This is a randomized, controlled, multicenter, phase II clinical trial, evaluating the efficacy and safety of aumolertinib plus apatinib versus aumolertinib as first-line treatment in patients with EGFR mutation positive locally advanced or metastatic non-small cell lung cancer (NSCLC). The participants will be randomly assigned (1:1) to receive either oral aumolertinib (110 mg/day) plus apatinib (250 mg/day) or oral aumolertinib alone (110 mg/day).

Study Process:
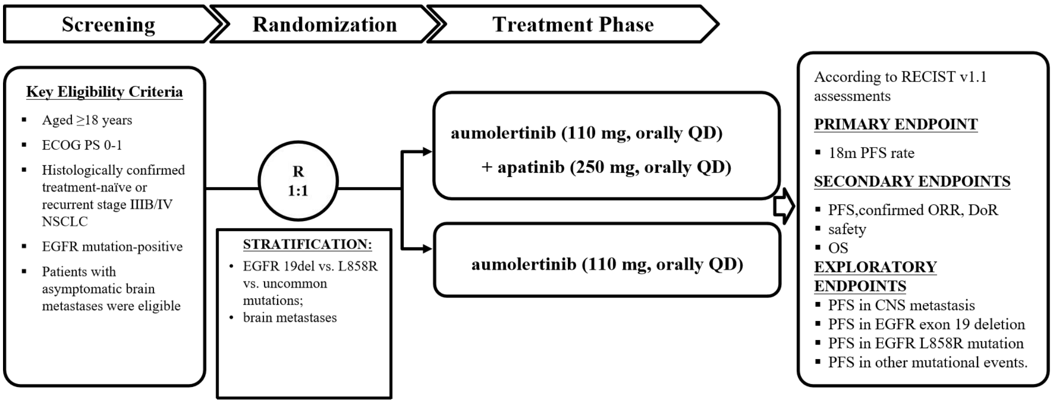
</td></tr>
<tr><td>Treatment Duration:
One study treatment cycle is defined as 21</td><td>Study duration:
Based on the study design, the study</td></tr>
</table>

days of continuous administration. Patients continuously receive treatment until the disease progresses or the discontinuation criteria are met.	will require at least 18 months
**Number of Subjects**:<br>Estimated number: Approx. 98 patients	**Number of Study Centers**:<br>Estimated number: Approx. 20 sites
**Dosage regimen**:<br>1. Aumolertinib tablets: Strength 55 mg/tablet; dose is 110 mg/day (2 tablets/day) by oral administration once daily.<br>2. Apatinib: Strength 250 mg/tablet; dose is 250 mg/day (1 tablet/day) by oral administration once daily.	
**Study Population**:<br>The patients are over 18 of age with stage IIIB–IV NSCLC confirmed by histological examination. The patients have not received any systemic therapies and are confirmed to have *EGFR* sensitizing mutations (exon 19 deletion, L858R, or other *EGFR* sensitive mutations) prior to enrollment.	
**Primary endpoint**:<br>18-month PFS rate	
**Secondary endpoint**:<br>PFS, confirmed ORR (the proportion of patients with confirmed complete response or partial response), DoR (time from the first documented response to the first time of disease progression or death), safety, and OS.	
**Exploratory endpoint:**<br>PFS benefits in patients with CNS metastasis, *EGFR* exon 19 deletion, *EGFR* L858R mutation, and other mutational events.	
**Inclusion Criteria**:<br>A subject must meet all the following inclusion criteria in order to be enrolled in this study:	

1. Age over 18 years.

2. An Eastern Cooperative Oncology Group (ECOG) performance status score of 0 or 1, with no worsening over the previous 2 weeks, and minimum expected survival of 12 weeks.

3. Histological or cytological confirmed locally advanced or metastatic NSCLC (including patients relapsing after prior surgical treatment or with an initial diagnosis of stage IIIB/IV, according to the AJCC 8th edition lung cancer staging criteria).

4. The patients have not received any systemic therapy after a confirmed diagnosis of locally advanced or metastatic NSCLC. For a patient who has received local treatment, if the lesions within the scope of the local treatment are non-target lesions, the patient may participate in the study.

5. Patients who have previously received preoperative neoadjuvant chemotherapy, postoperative adjuvant chemotherapy and concurrent chemoradiotherapy should meet the conditions that they have completed treatment for more than 6 months (inclusive 6 months).

6. Tumor tissue sample, blood sample, pleural effusion, peritoneal effusion or cerebrospinal fluid sample is tested by the central lab and confirmed positive for an *EGFR*-sensitizing mutation (Including but not limited to exon 19 deletion, or exon 21L858R, G719A, L861Q, etc.).

7. According to RECIST 1.1, participants must have measurable target lesions examined by PET-CT or CT or MRI. Imaging evaluation of the tumor will be performed within 28 days prior to the first dose.

8. Patients with asymptomatic brain metastases or brain lesions whose condition is stable after radiotherapy and who have completed radiotherapy for more than 28 days (including 28 days) can be enrolled.

9. Women of childbearing age must take appropriate contraceptive measures from screening to 3 months after the end of the study treatment and should not

breastfeed. Before administering the drugs, a negative pregnancy test or meeting one of the following criteria proving that there is no risk of pregnancy:

a) Post-menopause is defined as amenorrhea for at least 12 months after discontinuation of all exogenous hormone replacement therapy at an age older than 50 years.

b) Women under the age of 50 may also deemed to be post-menopausal if they have experienced amenorrhea for 12 or more months after discontinuation of all exogenous hormone therapy, and their luteinizing hormone (LH) and follicle-stimulating hormone (FSH) levels are within the laboratory reference range for post-menopause.

c) Having previously undergone irreversible sterilization, including hysterectomy, bilateral oophorectomy, or bilateral salpingectomy, but not including bilateral tubal ligation.

10. From screening to 3 months after the end of the study treatment, male patients should use barrier contraceptives (i.e. condoms).

11. Subjects are personally willing to take part and sign a written Informed Consent Form.

Exclusion Criteria:

A subject will not be eligible for inclusion in this study if any of the following criteria apply:

1. Receipt of any of the following treatments:
    a) Receipt of any *EGFR* tyrosine kinase inhibitor therapy in the past.
    b) Receipt of any VEGF receptor tyrosine kinase inhibitor or other anti-angiogenic agents;
    c) Receipt of any PD-1/PD-L1 inhibitor or other immune checkpoint inhibitor agents;
    d) The patient underwent major surgery within 4 weeks before the first dose of the study drug.

e) Receipt of radiotherapy for more than 30% of the bone marrow or extensive radiotherapy within 4 weeks before the first dose of the study drug.

f) Receipt of potent CYP3A4 inhibitors, inducers, or drugs with a narrow therapeutic window which are CYP3A4 sensitive substrates within 7 days before the first dose of the study drug.

2. Patients with symptomatic brain metastases.
3. Patients with other driver gene mutation including *ALK*/*ROS1* fusions.
4. Patients with other malignant tumors and requiring standard treatment or major surgery within 2 years after the first dose of the study treatment.
5. At the beginning of study treatment, those with residual toxicity from prior treatment greater than CTCAE grade 1 that cannot be alleviated, with the exception of hair loss and grade 2 neurotoxicity from prior chemotherapy.
6. Suspected active tuberculosis infection or acquired immunodeficiency syndrome antibody (Anti-HIV) positive, anti-treponema treponema antibody (TP-Ab) positive, Hepatitis B surface antigen (HBV-AG) positive, HBV DNA copy number > the upper limit of normal test unit and other serious active fungal, bacterial and/or viral infections.
7. Severe gastrointestinal dysfunction that may affect drug intake, transport, or absorption, such as inability to take oral drugs, uncontrollable nausea or vomiting, extensive gastrointestinal resection history, untreated recurrent diarrhea, atrophic gastritis, untreated stomach diseases requiring long-term use of proton pump inhibitors, Crohn's disease, ulcerative colitis, etc.
8. Hepatic encephalopathy, hepatorenal syndrome or cirrhosis.
9. Abnormal coagulation function (APTT > 1.5×ULN or INR > 1.5), thrombolysis, anticoagulation or antiplatelet therapy, bleeding tendency or active bleeding (such as hemoptysis, gastrointestinal bleeding, etc.); Arterial/venous thrombotic events such as cerebrovascular accidents (including

transient ischemic attacks), deep venous thrombosis, and pulmonary embolism occurred in the 6 months prior to study initiation.

10. Patients with hypertension who cannot be reduced to the normal range by antihypertensive medication (systolic blood pressure >140 mmHg, diastolic blood pressure >90 mmHg).
11. A wound or fracture that has not healed for a long time.
12. Urine routine indicates urinary protein ≥++, or 24-hour urinary protein quantity > 1.0g.
13. Grade 3-4 cardiac insufficiency (NYHA criteria) or meet any of the following cardiac test results:
    a) Mean corrected QT interval (QTc) of >470 msec from 3 resting electrocardiograms (ECG), using Fridericia's formula to conduct QT interval correction (QTcF).
    b) Various clinically significant, important abnormalities in rhythm, conduction, or ECG morphology in a resting ECG (such as complete left bundle branch block, 3rd degree atrioventricular block, 2nd degree atrioventricular block, and PR interval >250 msec).
    c) Any factors that increase the risk of QTc prolongation or arrhythmic events, such as heart failure, hypokalemia, congenital long QT syndrome, a history of long QT syndrome or sudden unexplained death under 40 years of age in the immediate family, or any concomitant drugs prolonging the QT interval.
    d) Left ventricular ejection fraction (LVEF) ≤50%.
14. Insufficient bone marrow reserve or organ function, reaching the following laboratory limits:
    a) Absolute neutrophil count $<1.5\times10^9/L$.
    b) Platelet count $<100\times10^9/L$.
    c) Hemoglobin <90 g/L (<9 g/dL).

d) If no demonstrable liver metastases, alanine aminotransferase >2.5 times the ULN; in the presence of liver metastases, alanine aminotransferase >5×ULN.

e) If no demonstrable liver metastases, aspartate aminotransferase >2.5×ULN; in the presence of liver metastases, aspartate aminotransferase >5×ULN.

f) If no demonstrable liver metastases, total bilirubin >1.5×ULN; in the presence of Gilbert's syndrome (unconjugated hyperbilirubinemia) or liver metastases, total bilirubin >3×ULN.

g) Creatinine >1.5×ULN and creatinine clearance <50 mL/min (calculated by the CockcroftGault formula); confirmation of creatinine clearance is only required when creatinine >1.5×ULN.

h) Serum albumin (ALB) <28 g/L.

15. History of interstitial lung disease, history of drug-induced interstitial lung disease, history of radiation pneumonitis requiring steroid therapy, or any evidence of clinically active interstitial lung disease.
16. History of hypersensitivity to any active or inactive ingredient of aumolertinib or to drugs with a similar chemical structure to or of the same class as aumolertinib; History of hypersensitivity to any active or inactive ingredient of apatinib or to drugs with a similar chemical structure to or of the same class as apatinib
17. Any severe or uncontrolled ocular disease (especially severe dry eye syndrome, dry keratoconjunctivitis, severe exposure keratitis, or other disease that may increase epithelial damage) that, in the physician's judgment, may increase the subject's safety risk; Or eye abnormalities that require surgery or are expected to require surgery during the study period.
18. Patients whose compliance with the procedures and requirements of the study could be poor, as determined by an investigator. If the subject has a clear

history of neurological or psychiatric disorders (including epilepsy or dementia), is currently suffering from mental disorders, etc., Patients in whom any conditions compromising patient safety or interfering with study assessments are present, as determined by an investigator.
**Efficacy Evaluation**:<br>According to RECIST 1.1 criteria for solid tumor efficacy, evaluating the efficacy of aumolertinib combined with apatinib versus aumolertinib alone in first-line treatment of advanced NSCLC subjects with *EGFR* sensitive mutations.<br>Efficacy endpoint:<br>1. Primary endpoint: 18-month PFS rate.<br>2. Secondary endpoints: PFS, confirmed ORR, DoR, safety, and OS.<br>3. Exploratory endpoints: PFS benefits in patients with CNS metastasis, *EGFR* exon 19 deletion, *EGFR* L858R mutation, and other mutational events.
**Safety Evaluation**:<br>Observe and record any adverse events experienced by subjects during the clinical trial period, including clinical symptoms, abnormal vital signs, and abnormal laboratory tests; record severity, time of onset, duration, treatment method, and prognosis; and determine its relatedness to the study drug. Safety endpoints: Occurrence of adverse events; occurrence of serious adverse events; proportion of dose modification, patients withdrawn or due to adverse events.
**Calculation of Sample Size**:<br>Given assumptions of 18-month PFS rate of 75% for aumolertinib plus apatinib and 50% for aumolertinib alone, a drop-out rate of 10%, at the significance level of 0.05, to achieve the power of 0.8, a total number of 98 subjects are needed (49 per arm).

3. Table of Abbreviations

AE	Adverse Event
ALT	Alanine aminotransferase
APTT	Activated partial thromboplastin time
AST	Aspartate aminotransferase
C1D1	Cycle 1 Day 1
CI	Confidence interval
CNS	central nervous system
CR	Complete response
CT	Computed tomography
CTCAE	Common Terminology Criteria for Adverse Events
CYP	Cytochrome P450
DCR	Disease control rate
DLT	Dose limiting toxicity
DNA	Deoxyribonucleic acid
DoR	Duration of response
ECG	Electrocardiogram
ECOG	Eastern Cooperative Oncology Group
EGFR	Epidermal growth factor receptor
EGFRm^{+}	Epidermal growth factor receptor sensitizing mutation positive
FAS	Full analysis set
FSH	Follicle-stimulating hormone
HIV	Human immunodeficiency virus
HR	Hazard ratio
IDMC	Independent Data Monitoring Committee
INR	International normalized ratio
LH	Luteinizing hormone
LVEF	Left ventricular ejection fraction

mPFS	Median progression-free survival
MRI	Magnetic resonance imaging
MTD	Maximum tolerated dose
NE	Not evaluable
NSCLC	Non-small cell lung cancer
ORR	Objective response rate
OR	Odds ratio
OS	Overall survival
PFS	Progression-free survival
PD	Progressive disease
PET-CT	Positron emission tomography and X-ray computed tomography system
PK	Pharmacokinetics
PR	Partial response
QD	Once daily
QT	ECG interval measured from the beginning of the QRS complex to the end of the T wave
QTc	Corrected QT interval
QTcF	Fridericia-corrected QTc
RECIST 1.1	Response Evaluation Criteria in Solid Tumors (version 1.1)
SAE	Serious adverse event
SD	Stable disease
T790M	Mutation of amino acid at position 790 in *EGFR* from threonine to methionine
$T790M^+$	T790M mutation positive
TL	Target lesion
TKI	Tyrosine kinase inhibitor

ULN	Upper limit of normal

4. Research Background and Scientific Basis

4.1 Background

Lung cancer is one of the most common malignancies in the world. In 2018, cancer data from China indicated that lung cancer had the highest incidence and mortality rates, accounting for more than 20%. Non-small cell lung cancer (NSCLC) accounts for 80%-85% of all lung cancers, approximately 70% of lung cancer patients are already at an advanced stage, either locally or with distant metastasis, by the time of initial diagnosis, making surgical resection unfeasible. Additionally, patients who initially undergo successful surgical resection later develop distant metastases by the time of relapse[1]. The median survival of patients with advanced NSCLC who cannot be surgically resected is often less than one year[2].

Epidermal growth factor receptor (*EGFR*) sensitive mutations (*EGFR*m+) are the most common driver gene mutations in NSCLC, accounting for 10%-17% of Western populations and 30%-50% of Asian populations [3,4]. Tyrosine kinase inhibitors (TKI) targeting *EGFR*-sensitive mutations have become the first-line standard treatment for locally advanced or metastatic NSCLC with *EGFR*m+, with progression-free survival (PFS) of 8-18.9 months [5-7]. But patients who initially respond to EGFR-TKIs will inevitably develop disease progression due to secondary resistance. Therefore, it is urgent to delay or overcome EGFR-TKIs resistance, improve patients' PFS and overall survival (OS), and explore new treatment options.

Vascular endothelial growth factor receptor (VEGFR) is a signaling pathway parallel to *EGFR* but intersecting downstream. VEGFR tyrosine kinase inhibitors inhibit the VEGFR signaling pathway, control tumor angiogenesis, and may play a role in EGFR-TKI resistance [8-10]. The combination of anti-angiogenic agents and EGFR-TKI, inhibiting both VEGFR and EGFR pathways, improves the response to

targeted therapy and delays the onset of acquired resistance in NSCLC patients.

Currently, the combination therapy of anti-angiogenic drugs and EGFR-TKI (also known as "A+T" combination therapy) has been shown to be effective in NSCLC. Domestic and foreign guidelines such as ESMO and CSCO have recommended "A+T" as the first-line treatment for *EGFR* driver gene positive in advanced NSCLC patients [5].

In February 2011, the JO25567 study (randomized, controlled, Phase II study) conducted in Japan, compared erlotinib plus bevacizumab versus erlotinib alone in the first-line treatment of 154 Japanese patients with advanced *EGFR*-sensitive mutated non-squamous NSCLC. The results showed that "A+T" significantly prolonged patients' PFS (16.0 months vs 9.7 months, P=0.0015) [11]. The subsequent NEJ026 study (randomized, controlled, Phase III study) enrolled 228 patients who received bevacizumab plus erlotinib or erlotinib alone. The results showed that PFS was 3.6 months longer in the combination group than in the monotherapy group (16.9 months vs 13.3 months, P=0.016)[12]. The RELAY study (global, double-blind, Phase III clinical trial) compared the efficacy and safety of erlotinib plus ramucirumab with erlotinib alone in patients with stage IV or post-operatively relapsed *EGFR* in 449 global participants. Results showed that the "A+T" group significantly extended patients' median progression-free survival (mPFS) by 7 months (19.4vs 12.4, P<0.0001) [13].

However, in the above combination study protocol, both bevacizumab and ramucirumab required intravenous administration, which undoubtedly increased the number of repeated admissions and visits of patients, and had a certain impact on the quality of life. The ACTIVE study combining apatinib with gefitinib is currently underway[14]. Dual-target oral "A+T" regimen provides patients with advanced NSCLC with more options and better quality of life. However, more high-quality evidence-based medical evidence is needed to support this approach.

Aumolertinib (HS10296) is a class 1 new drug independently developed by

Jiangsu Hansoh Pharmaceutical Group Co., LTD. The third generation of small molecule EGFR-TKI, which can irreversibly and highly selectively inhibit *EGFR* sensitive mutations (such as exon 19 deletion and L858R mutation) and Mutation of amino acid at position 790 in *EGFR* from threonine to methionine (T790M) resistant mutations. At the same time, the activity against wild-type *EGFR* is minimal, resulting in a low incidence of adverse reactions.

Apatinib (YN968D1) is a new targeted drug developed and produced in China, and the world's first effective and safe small-molecule anti-angiogenesis targeted drug for gastric cancer. A previous multicenter Phase II study of 105 patients with advanced NSCLC confirmed that Apatinib is efficacy and tolerable in pretreated patients with advanced NSCLC [15].

Therefore, this study aims to evaluate the efficacy and safety of aumolertinib plus apatinib versus aumolertinib alone in first-line treatment of advanced NSCLC with epidermal growth factor receptor-sensitive mutation (*EGFR*m+).

4.2 Scientific Evidence

Aumolertinib has been granted new drug clinical trial approvals by both the U.S. Food and Drug Administration and the National Medical Products Administration in mainland China and is currently undergoing international multicenter Phase I/II trials. The phase I study is an open, multicenter study in patients with locally advanced or metastatic NSCLC whose disease has progressed following prior EGFR-TKI treatment, including dose escalation and dose extension.

The single-dose Pharmacokinetics (PK) data of aumolertinib in advanced NSCLC patients who had progressed after previous EGFR-TKI treatment showed that aumolertinib was absorbed quickly after a single fasting oral administration, the blood concentration peaked at 4.0 hours, and the mean drug elimination half-life ranged from 30.7 to 37.5 hours. In the dose range of 55mg to 220 mg, the PK parameters C max and AUC of aumolertinib were linearly correlated with the dose. The exposure of active metabolite HAS-719 (major circulating metabolite of HS10296) was about 1/3

of that of the original drug, and the PK parameters showed a linear relationship. The peak time of HAS-719 was about 6 hours later than that of the original drug, and the half-life of the drug elimination was 50-70 hours. The PK test data showed that the plasma concentration of aumolertinib reached a stable state after 7 consecutive days of once-daily administration (QD). Compared with a single dose, there was almost no accumulation in steady state. In the dose range of 55mg to 220mg, when continuous administration reached homeostasis, aumolertinib exposure had a linear relationship with steady-state blood concentration and area under the curve.

A total of 26 patients were included in the phase I dose escalation trial of aumolertinib. No dose limiting toxicity (DLT) occurred in the low-dose group (55/110mg), only 1 of 6 patients in the 220mg dose group had DLT (grade 3 anemia), and only 1 of 6 patients in the 260mg dose group had DLT, grade 3 creatinine elevation; The maximum tolerated dose (MTD) was not reached in the dose escalation phase. Overall objective response rate (ORR) and disease control rate (DCR) were 52.4% and 85.7% for patients eligible for interim evaluation (n=21). In the 55mg (6 cases) and 110mg (6 cases) dose groups, the ORR was 66.7% and DCR was 100.0%. In the 220mg dose group (6 cases), ORR was 50.0%, DCR was 83.3%, and in the 260mg dose group, only 3 cases completed treatment response assessment, DCR was 33.3%。

The Phase I extension study of aumolertinib has completed enrollment in a T790M mutation-positive population, with a total of 91 participants enrolled in the 55mg (30 cases), 110mg (30 cases), and 220mg (31 cases) dose groups. In terms of safety, the common adverse events (AEs) was rash, fatigue, blurred vision, decreased white blood cells, gastrointestinal symptoms (such as constipation, diarrhea, nausea), and elevated levels of creatine kinase and alanine aminotransferase. However, symptoms were mild or asymptomatic, and most AEs resolved on their own. No drug-related serious AEs (SAEs) occurred in the 55mg and 110mg dose groups. In the 220mg dose group, the incidence and severity of AE increased significantly. In terms

of treatment response, among patients eligible for interim evaluation (n=76), overall ORR was 50.0% and DCR was 92.1%. In the 55mg dose group (30 cases), ORR was 53.3% and DCR was 83.3%. The ORR and DCR of 110mg (30 cases) were 46.7% and 96.7% respectively. In the 220mg dose group (16 cases), ORR was 50.0% and DCR was 100.0%.

The data of 55mg and 110mg in the dose escalation phase and the extension phase were combined for analysis: In the phase I clinical trial of the second-line treatment of advanced NSCLC, 36 patients with T790M mutation-positive were enrolled in the 55mg and 110mg dose groups respectively, and the last patient was followed up for 3 months after enrollment. All patients had been evaluated for efficacy at least twice. At present, there are no unintended safety concerns in either dose group. The overall ORR for T790M mutation was 52.1%, 55.6% in the 55mg dose group and 50.0% in the 110mg dose group. The overall DCR was 92.6%, including 86.1% in the 55mg dose group and 97.2% in the 110mg dose group.

The results of the Phase II single-arm clinical study of aumolertinib were announced at the 2019 World Congress on Lung Cancer (WCLC). The study enrolled 244 patients with locally advanced or metastatic NSCLC with *EGFR* T790M mutation, as well as asymptomatic BMS, and showed an ORR of 68.4%, DCR was 93.4%; The 12-month progression-free survival rate was 53%. Aumolertinib was well tolerated, and the most common adverse events were elevated of creatine phosphokinase, rash, pruritus, elevated of AST/ ALT, anemia, and decreased white blood cell count. Grade 3 or higher drug-related adverse events occurred in 20.9% patients, and no interstitial pneumonia was reported.

In addition, the randomized, double-blind, controlled phase III clinical study of aumolertinib versus gefitinib in first-line treatment of advanced NSCLC with *EGFR* mutations (NCT03849768) is ongoing, with enrollment of 350 participants completed in October 2019, and the primary endpoint reached statistically significant positive results. Specific data will also be presented at international academic conferences in

due course.

Aumolertinib ® (Aumolertinib mesylate tablet) is a class 1 innovative drug independently developed by Jiangsu Hansoh Pharmaceutical Group Co., LTD., and has been approved by the National Medical Products Administration (NMPA) on March 18 (approval number: H20200004) for the treatment of "adult subjects with locally advanced or metastatic NSCLC who have experienced disease progression during or after prior treatment with EGFR-TKI and have been tested positive for *EGFR* T790M mutation."

Based on the above research and the clinical data of aumolertinib, we designed this study to evaluate the efficacy and safety of aumolertinib plus apatinib versus aumolertinib alone in first-line treatment of advanced NSCLC with *EGFR*m+.

5. Study Endpoints

The endpoints of this study will be analyzed based on the tumor response assessment by investigator per Response Evaluation Criteria in Solid Tumors (version 1.1) (RECIST 1.1).

Primary endpoint is 18-month PFS rate.

Secondary endpoints consist of PFS, confirmed objective response rate (ORR, the proportion of patients with confirmed complete response or partial response), duration of response (DoR, time from the first documented response to the first time of disease progression or death), safety, and OS.

Exploratory endpoints consist of PFS benefits in patients with central nervous system (CNS) metastasis, *EGFR* ex19del, *EGFR* L858R mutation, and other mutational events.

6. Sample Size

This is a multicenter, randomized, open-label study with a superiority design. The primary endpoint is 18-month PFS rate. Based on the assumptions of 18-month PFS rate of 75% for aumolertinib plus apatinib and 50% for aumolertinib alone, a drop-out rate of 10%, at the significance level of 0.05, to achieve the power of 0.8, a

total number of 98 subjects are needed (49 per arm).

7. Study Design and Description

7.1 Study Design

This is a multi-center, randomized, open-label, phase II clinical trial to evaluate the efficacy and safety of aumolertinib plus apatinib vs. aumolertinib monotherapy as first-line treatment in subjects with epidermal growth factor receptor mutation-positive (*EGFR*m+) advanced NSCLC. Subjects who meet the eligibility criteria will be randomized to the aumolertinib + apatinib group (test group) or the aumolertinib monotherapy group (control group) in a 1:1 ratio. Subjects in the test group will receive oral administration of aumolertinib 110 mg and apatinib 250 mg once daily, and subjects in the control group will receive oral administration of aumolertinib 110 mg once daily, in order to compare the efficacy and safety of the two treatment regimens. The schematic overview of the study is shown in Figure 1.

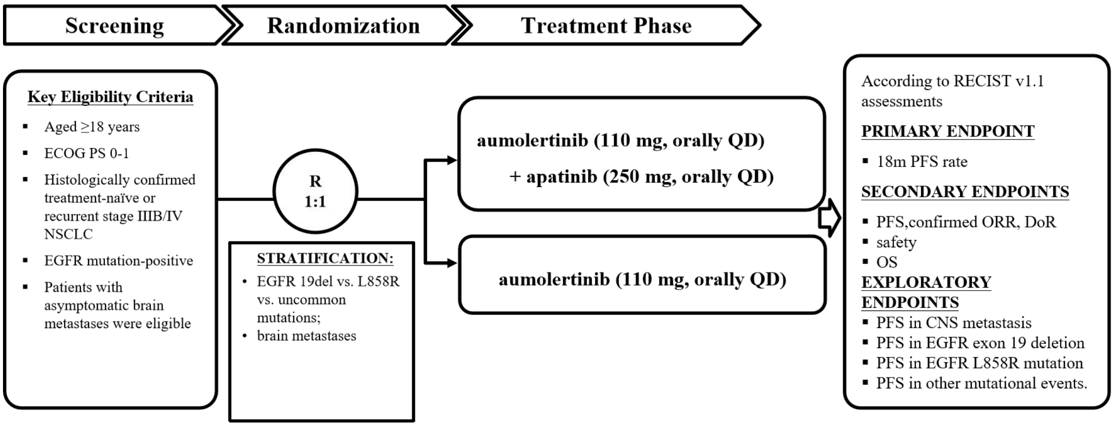

Figure 1. Schematic overview of the study

7.2 Inclusion Criteria

A subject must meet all the following inclusion criteria in order to be enrolled in this study:

1. Age over 18 years.
2. An Eastern Cooperative Oncology Group (ECOG) performance status score of 0 or 1, with no worsening over the previous 2 weeks, and minimum expected

survival of 12 weeks.

3. Histological or cytological confirmed locally advanced or metastatic NSCLC (including patients relapsing after prior surgical treatment or with an initial diagnosis of stage IIIB/IV, according to the AJCC 8th edition lung cancer staging criteria).
4. The patients have not received any systemic therapy after a confirmed diagnosis of locally advanced or metastatic NSCLC. For a patient who has received local treatment, if the lesions within the scope of the local treatment are non-target lesions, the patient may participate in the study.
5. Patients who have previously received preoperative neoadjuvant chemotherapy, postoperative adjuvant chemotherapy and concurrent chemoradiotherapy should meet the conditions that they have completed treatment for more than 6 months (inclusive 6 months).
6. Tumor tissue sample, blood sample, pleural effusion, peritoneal effusion or cerebrospinal fluid sample is tested by the central lab and confirmed positive for an *EGFR*-sensitizing mutation (Including but not limited to exon 19 deletion, or exon 21L858R, G719A, L861Q, etc.).
7. According to RECIST 1.1, participants must have measurable target lesions examined by PET-CT or CT or MRI. Imaging evaluation of the tumor will be performed within 28 days prior to the first dose.
8. Patients with asymptomatic brain metastases or brain lesions whose condition is stable after radiotherapy and who have completed radiotherapy for more than 28 days (including 28 days) can be enrolled.
9. Women of childbearing age must take appropriate contraceptive measures from screening to 3 months after the end of the study treatment and should not breastfeed. Before administering the drugs, a negative pregnancy test or meeting one of the following criteria proving that there is no risk of pregnancy:
   a) Post-menopause is defined as amenorrhea for at least 12 months after

discontinuation of all exogenous hormone replacement therapy at an age older than 50 years.

b) Women under the age of 50 may also deemed to be post-menopausal if they have experienced amenorrhea for 12 or more months after discontinuation of all exogenous hormone therapy, and their luteinizing hormone (LH) and follicle-stimulating hormone (FSH) levels are within the laboratory reference range for post-menopause.

c) Having previously undergone irreversible sterilization, including hysterectomy, bilateral oophorectomy, or bilateral salpingectomy, but not including bilateral tubal ligation.

10. From screening to 3 months after the end of the study treatment, male patients should use barrier contraceptives (i.e. condoms).
11. Subjects are personally willing to take part and sign a written Informed Consent Form.

7.3 Exclusion Criteria

Exclusion Criteria:

A subject will not be eligible for inclusion in this study if any of the following criteria apply:

1. Receipt of any of the following treatments:

a) Receipt of any *EGFR* tyrosine kinase inhibitor therapy in the past.

b) Receipt of any VEGF receptor tyrosine kinase inhibitor or other anti-angiogenic agents;

c) Receipt of any PD-1/PD-L1 inhibitor or other immune checkpoint inhibitor agents;

d) The patient underwent major surgery within 4 weeks before the first dose of the study drug.

e) Receipt of radiotherapy for more than 30% of the bone marrow or extensive radiotherapy within 4 weeks before the first dose of the study drug.

f) Receipt of potent CYP3A4 inhibitors, inducers, or drugs with a narrow therapeutic window which are CYP3A4 sensitive substrates within 7 days before the first dose of the study drug.

2. Patients with symptomatic brain metastases.
3. Patients with other driver gene mutation including *ALK*/*ROS1* fusions.
4. Patients with other malignant tumors and requiring standard treatment or major surgery within 2 years after the first dose of the study treatment.
5. At the beginning of study treatment, those with residual toxicity from prior treatment greater than CTCAE grade 1 that cannot be alleviated, with the exception of hair loss and grade 2 neurotoxicity from prior chemotherapy.
6. Suspected active tuberculosis infection or acquired immunodeficiency syndrome antibody (Anti-HIV) positive, anti-treponema treponema antibody (TP-Ab) positive, Hepatitis B surface antigen (HBV-AG) positive, HBV DNA copy number > the upper limit of normal test unit and other serious active fungal, bacterial and/or viral infections.
7. Severe gastrointestinal dysfunction that may affect drug intake, transport, or absorption, such as inability to take oral drugs, uncontrollable nausea or vomiting, extensive gastrointestinal resection history, untreated recurrent diarrhea, atrophic gastritis, untreated stomach diseases requiring long-term use of proton pump inhibitors, Crohn's disease, ulcerative colitis, etc.
8. Hepatic encephalopathy, hepatorenal syndrome or cirrhosis.
9. Abnormal coagulation function (APTT > 1.5×ULN or INR > 1.5), thrombolysis, anticoagulation or antiplatelet therapy, bleeding tendency or active bleeding (such as hemoptysis, gastrointestinal bleeding, etc.); Arterial/venous thrombotic events such as cerebrovascular accidents (including transient ischemic attacks), deep venous thrombosis, and pulmonary embolism occurred in the 6 months prior to study initiation.
10. Patients with hypertension who cannot be reduced to the normal range by

antihypertensive medication (systolic blood pressure >140 mmHg, diastolic blood pressure >90 mmHg).

11. A wound or fracture that has not healed for a long time.

12. Urine routine indicates urinary protein ≥++, or 24-hour urinary protein quantity > 1.0g.

13. Grade 3-4 cardiac insufficiency (NYHA criteria) or meet any of the following cardiac test results:

   a) Mean corrected QT interval (QTc) of >470 msec from 3 resting electrocardiograms (ECG), using Fridericia's formula to conduct QT interval correction (QTcF).

   b) Various clinically significant, important abnormalities in rhythm, conduction, or ECG morphology in a resting ECG (such as complete left bundle branch block, 3rd degree atrioventricular block, 2nd degree atrioventricular block, and PR interval >250 msec).

   c) Any factors that increase the risk of QTc prolongation or arrhythmic events, such as heart failure, hypokalemia, congenital long QT syndrome, a history of long QT syndrome or sudden unexplained death under 40 years of age in the immediate family, or any concomitant drugs prolonging the QT interval.

   d) Left ventricular ejection fraction (LVEF) ≤50%

14. Insufficient bone marrow reserve or organ function, reaching the following laboratory limits:

   a) Absolute neutrophil count $<1.5\times10^9$/L.

   b) Platelet count $<100\times10^9$/L.

   c) Hemoglobin <90 g/L (<9 g/dL).

   d) If no demonstrable liver metastases, alanine aminotransferase >2.5 times the ULN; in the presence of liver metastases, alanine aminotransferase >5×ULN.

   e) If no demonstrable liver metastases, aspartate aminotransferase >2.5×ULN; in the presence of liver metastases, aspartate aminotransferase >5×ULN.

f) If no demonstrable liver metastases, total bilirubin >1.5×ULN; in the presence of Gilbert's syndrome (unconjugated hyperbilirubinemia) or liver metastases, total bilirubin >3×ULN.

g) Creatinine >1.5×ULN and creatinine clearance <50 mL/min (calculated by the CockcroftGault formula); confirmation of creatinine clearance is only required when creatinine >1.5×ULN.

h) Serum albumin (ALB) <28 g/L.

15. History of interstitial lung disease, history of drug-induced interstitial lung disease, history of radiation pneumonitis requiring steroid therapy, or any evidence of clinically active interstitial lung disease.

16. History of hypersensitivity to any active or inactive ingredient of aumolertinib or to drugs with a similar chemical structure to or of the same class as aumolertinib; History of hypersensitivity to any active or inactive ingredient of apatinib or to drugs with a similar chemical structure to or of the same class as apatinib.

17. Any severe or uncontrolled ocular disease (especially severe dry eye syndrome, dry keratoconjunctivitis, severe exposure keratitis, or other disease that may increase epithelial damage) that, in the physician's judgment, may increase the subject's safety risk; Or eye abnormalities that require surgery or are expected to require surgery during the study period.

18. Patients whose compliance with the procedures and requirements of the study could be poor, as determined by an investigator. If the subject has a clear history of neurological or psychiatric disorders (including epilepsy or dementia), is currently suffering from mental disorders, etc., Patients in whom any conditions compromising patient safety or interfering with study assessments are present, as determined by an investigator.

7.4 Randomization

This is a randomized, controlled, open-label, multicenter clinical trial. In this trial, randomization will be done by stratified blocked randomization (block size: 4)

using Clinflash IRT (Clinflash Healthcare Technology (Jiaxing) Co. Ltd., Zhejiang, People's Republic of China). The subjects will be randomized in a 1:1 ratio to the aumolertinib + apatinib group or the aumolertinib monotherapy group with *EGFR* mutation status (exon 19 deletion *vs.* exon 21 L858R *vs.* atypical mutations) and metastatic site (CNS metastasis *vs.* no CNS metastasis) at enrollment as stratification factors.

Eligible subjects will be assigned a unique randomization number on the day of the first dose of the investigational medicinal product (the first day of the first treatment cycle, C1D1). The investigator will assign a randomization number and medication number to all screened eligible subjects who qualify for the study after randomization.

8. Study Procedures

Each subject will be assessed by the same investigator or study site personnel when possible at each step.

The subject must read and voluntarily sign the ICF approved by the site's ethics committee prior to the initiation of any study procedures. All study procedures should be performed within the time windows indicated in the schedule of activities. See Appendix A for the overall follow-up schedule.

8.1 Screening Period

Inpatient or outpatient subjects will be screened after signing the informed consent form and undergo relevant laboratory tests and assessments. Subjects who meet all the inclusion criteria and do not meet any of the exclusion criteria may be enrolled.

8.2 Treatment Period

All eligible subjects will be randomized in a 1:1 ratio to the following 2 groups:

1) Aumolertinib + apatinib group:

Aumolertinib: strength: 55 mg/tablet; dose: 110 mg/day (2 tablets/day), p.o. QD;

Apatinib: strength: 250 mg/tablet; dose: 250 mg/day (1 tablet/day), p.o. QD.

2) Aumolertinib monotherapy group:

Aumolertinib: strength: 55 mg/tablet; dose: 110 mg/day (2 tablets/day), p.o. QD;

Each treatment cycle consists of 21 days of continuous administration. After treatment is started, efficacy will be evaluated once every 6 weeks (± 7 days) as per RECIST 1.1. Subjects will continue treatment until progressive disease assessed by investigator per RECIST 1.1 or until the withdrawal or discontinuation criteria are met. Subjects may continue to receive the treatment even if progressive disease per RECIST 1.1 occurs as long as they still benefit from the treatment as judged by investigator; if it is decided to continue the treatment, the baseline and follow-up plan will not be affected by the additional courses of treatment. Once the treatment is discontinued due to lack of clinical benefit upon judgment, it cannot be started again.

8.3 Follow-up Period

Subjects who discontinue the treatment due to meeting the discontinuation criteria should complete the treatment discontinuation follow-up, the 30 days safety follow-up after treatment discontinuation, and survival follow-up once every 12 weeks (± 7 days) as specified in the study plan.

Subjects without progressive disease at treatment discontinuation will be followed up once every 6 weeks (± 7 days) until progressive disease assessed by investigator per RECIST 1.1 (both intracranial and systemic), even if the subject has received other anti-tumor treatments; after progressive disease, the subjects will be followed up for survival once every 12 weeks as specified in the study plan.

9. Endpoint Evaluation and Statistical Analysis

9.1 Anti-Tumor Activity Evaluation

9.1.1 Investigator Assessment as per RECIST 1.1

At each visit, the investigator will assess the subject's tumor response according to RECIST 1.1 by comparing with baseline and previous assessments, depending on subject's status. The progression of target lesion (TL) will be calculated by comparing

with tumor burden at its nadir (i.e., the smallest sum of previously recorded diameters). In the absence of progression, tumor response and stable disease (SD) will be calculated by comparing with baseline tumor measurements obtained prior to the start of the treatment. For subjects with indeterminable tumor status, if there is evidence of progression, they will be considered to have progressive disease (PD); if there is no evidence of progression, they will be considered not evaluable (NE). The endpoints of this study will be analyzed based on the tumor response assessment by the investigator per RECIST 1.1, and the investigator will also manage subjects only based on the assessment results.

The investigator will evaluate the response to treatment using RECIST 1.1 by determining the ORR, DCR, DoR, and progression-free survival (PFS). In addition to the overall tumor imaging evaluation performed by the investigator as per RECIST 1.1, for extracranial and/or intracranial lesions (no more than 5 target lesions in the whole body) selected for overall evaluation, the respective intracranial and extracranial response evaluation (ORR, DCR, DOR) will be performed according to the response evaluation criteria in RECIST 1.1, and the investigator does not need to select additional lesions.

9.1.2 18-month Progression-free Survival (PFS) Rate

PFS is defined as the time from randomization to the first time of disease progression (assessed by RECIST v1.1) or death, whichever occurred earlier, evaluated by investigators. Patients with treatment discontinuation for any reason will be censored at the date of the last progression assessment with no documented progression. PFS-derived data are based on assessment date rather than visit date.

9.1.3 Overall survival (OS)

OS is defined as the time from the date of the first dose to the date of the subject's death due to any cause. A subject who has not died at the time of statistical analysis will be censored at the time point when the subject is last known to be alive.

9.1.4 Confirmed Objective Response Rate (ORR)

As per RECIST 1.1, confirmed ORR is defined as the percentage of subjects with complete response (CR) or partial response (PR) lasting for at least 2 times evaluation before PD.

Data until PD or last evaluable data in the absence of PD will be recorded in the assessment of ORR. However, any CR or PR that occurs after discontinuation of the study treatment and further anti-tumor treatment will not be included in the calculation of ORR.

Table 2 provides the criteria used to determine objective tumor response of TLs.

Table 2. Overall response of target lesions

**CR**	All TLs disappear since baseline. The short axis of any pathological lymph nodes selected as TLs must be less than 10 mm.
**PR**	The sum of diameters of TLs decreases by at least 30% from baseline.
**SD**	Neither sufficient shrinkage to qualify for PR nor sufficient increase to qualify for PD.
**PD**	The sum of diameters of TLs increases by at least 20% compared with the minimum diameter in the trial (including the baseline sum, if this is the minimum value in the trial). In addition to a relative increase of 20%, the sum must also show an absolute increase of at least 5 mm.
**NE**	Only relevant TLs are not assessed or not evaluable or have lesion intervention.<br>Note: If the sum of diameters meets the criteria of PD, the lesion is not evaluable and should be classified as a PD.

9.1.5 Duration of Response (DoR)

DoR is defined as the time from the date of first compliance with response to the date of PD or death due to any cause. The end date of the response should be

consistent with the date of the endpoint PFS. The start of DoR is defined as the latest date of the visit at which PR or CR criteria are first met. If a subject does not progress after a response, the censored time for PFS will be used for DoR.

9.2 Safety Evaluation

All safety data will be summarized at the end of the study. All data from the initiation of treatment until 30 days after the last dose will be summarized together in the safety evaluation. Data prior to initiation of treatment will be summarized but not included in the summary table of AEs.

9.3 Recommended Toxicity Management

During toxicity management, the dose reduction for adverse events of apatinib will be permitted to 250 mg every other day. If a subject still cannot tolerate the treatment despite receiving the lowest dose, it is recommended to discontinue the study treatment.

10. Statistical Analytical Methods

To assess the between-group differences, we will use (i) the Chi-square test for categorical variables, (ii) the Mann-Whitney test for continuous variables, (iii) the Kaplan-Meier method and the Log-rank test for time-to-event variables, and (iv) the "naïve" test based on the comparison of Kaplan-Meier estimators for the 18-month PFS rate ("Fixpoint.test" function of the R package: ComparisonSurv). Hazard ratios (HRs) and corresponding 95% confidence intervals (CIs) will be estimated using the Cox proportional hazards model. The 95% CIs for ORR will be generated through the Clopper-Pearson method. Univariable and multivariable analyses of PFS and ORR will be performed with the Cox proportional hazards model and the logistic regression model, respectively. The interaction effect between treatment and each key variable will be calculated using a Cox proportional hazards model (for PFS) or a logistic regression model (for ORR) that contains the treatment group, key variable, and treatment-by-variable interaction term. All statistical analyses mentioned above will

be performed using IBM SPSS Statistics 22 or R 4.1.3.

10.1 Efficacy Analysis

Efficacy endpoints will be analyzed in Full analysis set (FAS) using results evaluated by the investigator as per RECIST 1.1.

10.1.1 Analysis of Primary Efficacy Endpoint (18-month PFS rate)

The difference in 18-month PFS rate between the two arms will be assessed with the "naïve" test based on the comparison of Kaplan-Meier estimators ("Fixpoint.test" function of the R package: ComparisonSurv).

10.1.2 Analysis of Secondary Efficacy Endpoints

a) Analysis of Systemic PFS

The difference in PFS between the two arms will be evaluated with the Log-rank test. The HR and its 95% CI will be calculated using the Cox proportional hazards model.

b) Analysis of ORR

The difference in ORR between the two arms will be assessed with the Chi-square test. The 95% CIs for ORR will be generated through the Clopper-Pearson method.

c) Analysis of DoR

The difference in DoR between the two arms will be evaluated with the Log-rank test. The HR and its 95% CI will be calculated using the Cox proportional hazards model.

10.1.3 Analysis of Exploratory Endpoints

The PFS benefits in patients with CNS metastasis, *EGFR* exon 19 deletion, *EGFR* L858R mutation, and other mutational events will be estimated using the Log-rank test. The HR and its 95% CI will be calculated using the Cox proportional hazards model.

10.1.4 Time of Interim Analysis

The interim analysis will be initiated after all patients undergoing treatment have been followed up for over 18 months.

10.1.5 Time of Final Analysis

The final analysis for OS will be conducted after 59 death events (data maturity, 60%).

10.2 Safety Analysis

10.2.1 Adverse Events

The AEs that occur from the initiation of treatment until 30 days after the last dose will be summarized in the safety evaluation. The AEs that occur prior to initiation of treatment and AEs that occur after 30 days following the last dose will be tabulated separately and not included in the summary table of AEs.

Treatment-emergent AEs will be summarized by treatment group, mainly including:

1) Number and proportion of subjects with at least one AE;
2) Number and proportion of subjects with at least one treatment-related AE;
3) Number and proportion of subjects with at least one severe (CTCAE Grade ≥ 3) AE;
4) Number and proportion of subjects with at least one severe (CTCAE Grade ≥ 3) treatment-related AE;
5) Number and proportion of subjects with at least one SAE;
6) Number and proportion of subjects with AEs resulting in dose modification;
7) Number and proportion of subjects with AEs resulting in withdrawal;
8) Number and proportion of subjects with AEs resulting in death.

All AEs will be summarized by system organ classes and preferred term. All AEs will be tabulated.

10.2.2 Independent Data Monitoring Committee (IDMC)

This study will establish an Independent Data Monitoring Committee (IDMC). In the data review meeting of interim analysis, the safety and efficacy of subjects will be evaluated. At the same time, the IDMC will suggest the time point for the final analysis and provide formal recommendations regarding whether to continue or

terminate the study. This committee will include 2 independent oncologists, 1 independent statistician, and 1 chairman. The IDMC review meeting will be held periodically according to the charter of the IDMC. The study enrollment will continue during IDMC meetings. After the data review, the IDMC will provide suggestions on the time point of the final analysis, whether to continue the study, whether to modify the protocol or whether to discontinue the study.

References

1. Pisters KM, Le Chevalier T. Adjuvant chemotherapy in completely resected non-small-cell lung cancer. *J Clin Oncol*. May 10 2005;23(14):3270-8. doi:10.1200/jco.2005.11.478
2. Bonomi PD. Implications of key trials in advanced nonsmall cell lung cancer. *Cancer*. Mar 1 2010;116(5):1155-64. doi:10.1002/cncr.24815
3. Gahr S, Stoehr R, Geissinger E, et al. EGFR mutational status in a large series of Caucasian European NSCLC patients: data from daily practice. *Br J Cancer*. Oct 1 2013;109(7):1821-8. doi:10.1038/bjc.2013.511
4. Siegel RL, Miller KD, Jemal A. Cancer statistics, 2020. *CA Cancer J Clin*. Jan 2020;70(1):7-30. doi:10.3322/caac.21590
5. Ettinger DS, Wood DE, Aggarwal C, et al. NCCN Guidelines Insights: Non-Small Cell Lung Cancer, Version 1.2020. *J Natl Compr Canc Netw*. Dec 2019;17(12):1464-1472. doi:10.6004/jnccn.2019.0059
6. Maemondo M, Inoue A, Kobayashi K, et al. Gefitinib or chemotherapy for non-small-cell lung cancer with mutated EGFR. *N Engl J Med*. Jun 24 2010;362(25):2380-8. doi:10.1056/NEJMoa0909530
7. Soria JC, Ohe Y, Vansteenkiste J, et al. Osimertinib in Untreated EGFR-Mutated Advanced Non-Small-Cell Lung Cancer. *N Engl J Med*. Jan 11 2018;378(2):113-125. doi:10.1056/NEJMoa1713137
8. Scott AJ, Messersmith WA, Jimeno A. Apatinib: a promising oral antiangiogenic agent in the treatment of multiple solid tumors. *Drugs Today (Barc)*. Apr 2015;51(4):223-9. doi:10.1358/dot.2015.51.4.2320599
9. Tian S, Quan H, Xie C, et al. YN968D1 is a novel and selective inhibitor of vascular endothelial growth factor receptor-2 tyrosine kinase with potent activity in vitro and in vivo. *Cancer Sci*. Jul 2011;102(7):1374-80. doi:10.1111/j.1349-7006.2011.01939.x
10. Mi YJ, Liang YJ, Huang HB, et al. Apatinib (YN968D1) reverses multidrug resistance by inhibiting the efflux function of multiple ATP-binding cassette transporters. *Cancer Res*. Oct 15 2010;70(20):7981-91. doi:10.1158/0008-5472.Can-10-0111
11. Seto T, Kato T, Nishio M, et al. Erlotinib alone or with bevacizumab as

first-line therapy in patients with advanced non-squamous non-small-cell lung cancer harbouring EGFR mutations (JO25567): an open-label, randomised, multicentre, phase 2 study. *Lancet Oncol*. Oct 2014;15(11):1236-44. doi:10.1016/s1470-2045(14)70381-x

12. Saito H, Fukuhara T, Furuya N, et al. Erlotinib plus bevacizumab versus erlotinib alone in patients with EGFR-positive advanced non-squamous non-small-cell lung cancer (NEJ026): interim analysis of an open-label, randomised, multicentre, phase 3 trial. *Lancet Oncol*. May 2019;20(5):625-635. doi:10.1016/s1470-2045(19)30035-x

13. Nakagawa K, Garon EB, Seto T, et al. Ramucirumab plus erlotinib in patients with untreated, EGFR-mutated, advanced non-small-cell lung cancer (RELAY): a randomised, double-blind, placebo-controlled, phase 3 trial. *Lancet Oncol*. Dec 2019;20(12):1655-1669. doi:10.1016/s1470-2045(19)30634-5

14. Zhang Z, Luo F, Zhang Y, et al. The ACTIVE study protocol: apatinib or placebo plus gefitinib as first-line treatment for patients with EGFR-mutant advanced non-small cell lung cancer (CTONG1706). *Cancer Commun (Lond)*. Nov 7 2019;39(1):69. doi:10.1186/s40880-019-0414-4

15. Zhou C, Wang Y, Zhao J, et al. Efficacy and Biomarker Analysis of Camrelizumab in Combination with Apatinib in Patients with Advanced Nonsquamous NSCLC Previously Treated with Chemotherapy. *Clin Cancer Res*. Mar 1 2021;27(5):1296-1304. doi:10.1158/1078-0432.Ccr-20-3136

Appendix A Study Plan

● **Randomized treatment study plan**								
**Visit**	**Screening period**	**Treatment period**			**EOT follow-up**	**Follow-up period**		
	**1**	**2**	**3-7**	**8+**		**28-day follow-up**	**Progression follow-up once every 6 weeks**	**Survival follow-up once every 3 months**
**Treatment cycle/Day**		**C1 D1**	**C2-C6 D1**	**C7 +, every 6 weeks D1**	**NA**	**NA**	**NA**	**NA**
**Day**	**-28**	**1**	**22-126**	**127+**	**NA**	**NA**	**NA**	**NA**
**Time window (days)**	**NA**	**0**	**± 7**	**± 7**	**± 7**	**± 7**	**± 7**	**± 7**
Informed consent	╳							
Demographic and baseline characteristics	╳							
Medical/Surgical history	╳							
Inclusion/Exclusion criteria	╳							
EGFR mutation detection	╳							

- **Randomized treatment study plan**

Visit	Screening period	Treatment period			EOT follow-up	Follow-up period		
	1	2	3-7	8+		28-day follow-up	Progression follow-up once every 6 weeks	Survival follow-up once every 3 months
**Treatment cycle/Day**		**C1 D1**	**C2-C6 D1**	**C7 +, every 6 weeks D1**	**NA**	**NA**	**NA**	**NA**
**Day**	**-28**	**1**	**22-126**	**127+**	**NA**	**NA**	**NA**	**NA**
**Time window (days)**	**NA**	**0**	**± 7**	**± 7**	**± 7**	**± 7**	**± 7**	**± 7**
Physical examination (including body weight)	×	×	×	×	×			
Height	×							
ECOG PS Score	×	×	×	×	×		×	
Pregnancy test	×							
Vital signs	×	×	×	×	×			
Clinical biochemistry/Hematology/Urinalysis	×	×	×	×	×			

● **Randomized treatment study plan**								
**Visit**	**Screening period**	**Treatment period**			**EOT follow-up**	**Follow-up period**		
	**1**	**2**	**3-7**	**8+**		**28-day follow-up**	**Progression follow-up once every 6 weeks**	**Survival follow-up once every 3 months**
**Treatment cycle/Day**		**C1 D1**	**C2-C6 D1**	**C7 +, every 6 weeks D1**	**NA**	**NA**	**NA**	**NA**
**Day**	**-28**	**1**	**22-126**	**127+**	**NA**	**NA**	**NA**	**NA**
**Time window (days)**	**NA**	**0**	**± 7**	**± 7**	**± 7**	**± 7**	**± 7**	**± 7**
ECG	×	×	×	×	×			
Creatine phosphokinase（CK）	×	as clinically necessary						
Echocardiography (LVEF)	×	Once every 12 weeks beginning with the first dose, and when clinically needed			×			
Tumor assessment (RECIST v1.1)	×	Once every 6 weeks after randomization						
CNS assessment (RECIST v1.1)	×	Once every 6 weeks after randomization in patients with CNS metastases						

- **Randomized treatment study plan**

**Visit**	**Screening period**	**Treatment period**			**EOT follow-up**	**Follow-up period**		
	**1**	**2**	**3-7**	**8+**		**28-day follow-up**	**Progression follow-up once every 6 weeks**	**Survival follow-up once every 3 months**
**Treatment cycle/Day**		**C1 D1**	**C2-C6 D1**	**C7 +, every 6 weeks D1**	**NA**	**NA**	**NA**	**NA**
**Day**	**-28**	**1**	**22-126**	**127+**	**NA**	**NA**	**NA**	**NA**
**Time window (days)**	**NA**	**0**	**± 7**	**± 7**	**± 7**	**± 7**	**± 7**	**± 7**
Aumolertinib administration		× 110 mg，QD						
Apatinib administration		× 250 mg，QD						
Concomitant medication	←→						× 30 days of follow-up	
Adverse event	←→							
Anti-tumor treatment	×						×	×
Subsequent response /progression data							×	

● **Randomized treatment study plan**								
**Visit**	**Screening period**	**Treatment period**			**EOT follow-up**	**Follow-up period**		
	**1**	**2**	**3-7**	**8+**		**28-day follow-up**	**Progression follow-up once every 6 weeks**	**Survival follow-up once every 3 months**
**Treatment cycle/Day**		**C1 D1**	**C2-C6 D1**	**C7 +, every 6 weeks D1**	**NA**	**NA**	**NA**	**NA**
**Day**	**-28**	**1**	**22-126**	**127+**	**NA**	**NA**	**NA**	**NA**
**Time window (days)**	**NA**	**0**	**± 7**	**± 7**	**± 7**	**± 7**	**± 7**	**± 7**
Survival status								×
